# A revisit of farm size and productivity: Empirical evidence from a wide range of farm sizes in Nigeria

**DOI:** 10.1016/j.worlddev.2021.105592

**Published:** 2021-10

**Authors:** Oluwatoba J. Omotilewa, T.S. Jayne, Milu Muyanga, Adebayo B. Aromolaran, Lenis Saweda O. Liverpool-Tasie, Titus Awokuse

**Affiliations:** a African Development Bank (AfDB) Abidjan, Cote d’Ivoire; b Michigan State University, East Lansing, MI, USA; c Adekunle Ajasin University, Ondo State, Nigeria

**Keywords:** Inverse relationship, Agricultural productivity, Large & medium-scale farms, Farm size, Nigeria, Sub-Saharan Africa (SSA)

## Abstract

The relationship between farm size and productivity has been studied extensively in the agricultural and development economics literature. However, most of the documented evidence in sub-Saharan Africa (SSA) is based on samples of small-scale farms operating 5 ha or less, with very little evidence assessing this relationship over a wider range of farm sizes. This omission is especially important considering the rapid expansion of medium-scale farms in much of Africa. This study examines the farm size-productivity relationship over a range of farms between zero and 40 ha in Nigeria. It also tests whether there is heterogeneity in productivity within medium-scale farms depending on how they came into being. Using four measures of productivity, empirical estimates reveal a U-shaped relationship where the IR holds between zero and about 22 ha, turning positive afterwards. Moreover, when medium-scale farms are distinguished between those who were actively engaged as small-scale farmers and stepped up/expanded their scale of operation and those who were primarily in non-farm employment and later stepped into medium-scale farming, the turning point for farmers who stepped up into medium-scale farming is at 11 ha, in contrast to 22 ha for those who stepped in. Further evidence suggests heterogeneity in productivity within medium-scale farms depending on whether the owner-operators stepped up or stepped into medium-scale farming. These findings imply that policies facilitating smallholders’ ability to expand the scale of their activities could contribute substantially to growth in farm productivity, agricultural commercialization and increase in food security in Nigeria, although in most areas only a small proportion of smallholder households are in a position to do this.

## Highlights

• Inverse farm size-productivity relationship (IR) has been documented in Africa, but mostly among smallholders.
• We examine this IR over a wider range of farm sizes and among medium-scale farms (MSF) up to 40 ha in Nigeria.
• We find a U-shaped relationship with IR existing up to 22 ha (turning point) but productivity increases afterwards.
• Turning point for farmers who stepped up into MSFs is at 11 ha, in contrast to 22 ha for those who stepped in.
• Evidence indicates MSFs exhibit heterogeneity in productivity depending on their mode of entry into farming.

## 1 Introduction

The inverse relationship (IR) between farm size and farm productivity is a longstanding empirical regularity in agricultural and development economics literature. Starting with Chayanov (1926) in Russia and later established by Sen (1966) in India, this relationship has also been widely observed in sub-Saharan Africa (SSA) but mostly among farmers cultivating farms 5 hectares (ha) and below (e.g., Barrett, 1996, Carletto et al., 2013, Carletto et al., 2015, Julien et al., 2019). If smallholders are indeed more productive, then efforts to reserve as much of SSA’s potentially available cropland as possible for smallholder-led farms might promote farm productivity objectives (Hazell et al., 2010). Smallholder-led development has also been considered to be more inclusive, leading to more equitable forms of rural development with stronger growth linkages effects with the rest of the economy (Hazell et al., 2010, Johnston and Kilby, 1975, Mellor, 1995).

However, the rapid growth of medium-scale farms in many parts of Africa (Jayne et al., 2016) and recent efforts to promote commercial agriculture in Africa (African Development Bank, 2017) may intensify the farm size-productivity debate on the continent. Farms operating between 5 and 100 hectares account for 30 percent or more of national cultivated land in many African countries for which data is available (e.g., see Tables A2 and A3 of Lowder et al., 2019; Jayne et al., 2019). The fundamental question remains whether African agricultural development and food security can be most effectively achieved mainly by prioritizing and promoting small-scale farm holdings or if alternative modes or scales of production are required (Collier & Dercon, 2014). Evidence to guide such policy discussion requires empirical evaluation of the farm size-productivity relationship across a much wider range of farm sizes than is available from most IR studies of SSA. Consequently, we are not so interested in whether one-hectare farms are more or less productive than 3-hectare farms, but rather whether one-hectare farms are more or less productive than farms of 30, 300, or 3000 ha.

Addressing these questions has been almost impossible in SSA given the paucity of detailed survey data on medium and especially large-scale farms.^1^ To our knowledge, Muyanga and Jayne (2019) is the only study to have examined this relationship over a relatively wide range of farms in SSA, but even this study only considers farms up to 70 ha. Examining this relationship in Kenya, the authors not only confirmed what has been mostly observed in the literature that IR exists on farm sizes 3 ha and below, but find a positive relationship between productivity and farm size between 5 and 70 ha.

Like Muyanga and Jayne (2019), our objective is to examine farm size-productivity relationships across a relatively wide range of farm sizes ranging up to 40 ha using data from two representative states in Nigeria, thereby providing the first empirical assessment of this issue in West Africa. However, this study builds upon Muyanga and Jayne (2019) by testing whether the productivity of medium-scale farms, and hence the relationship between farm size and productivity, systematically differs according to the mode of entry of farm operators into farming. Prior evidence on the characteristics of medium-scale farmers points to two distinct entry pathways: (i) those whose primary source of employment was small-scale farming operating <5 ha of land, who acquired additional land and expanded their operations into medium-scale farming; and (ii) individuals primarily engaged in off-farm employment who subsequently acquired land and started farming between 5 and 100 ha of land (Jayne et al., 2016, Jayne et al., 2019).

This paper makes three key contributions to the IR literature. First, using new primary data, we examine the farm size-productivity relationship across a wider range of farm sizes than virtually all other African studies (except for Muyanga & Jayne, 2019). The study provides an additional check on the robustness of Muyanga and Jayne’s findings of a U-shaped relationship between farm size and productivity. Understanding this relationship may be important for guiding policy decisions on land allocation and public agricultural expenditure priorities. Second, we extend Muyanga and Jayne (2019) which assumed homogeneity among medium-scale farm operators and explore potential heterogeneity in productivity among medium-scale farms. More specifically, we distinguish between the modes of entry into medium-scale farms as follows: i) medium-scale farm operators who were originally small-scale producers and who subsequently expanded their landholdings and farm operations, *stepping-up* into medium-scale farming, vs. ii) those who *stepped into* medium-scale farming, who are likely commercial investors in agriculture, with limited prior involvement in farming.^2^ Third, we use multiple measures of productivity. Rather than restrict productivity to nominal measures of a single crop production or output per hectare (yield) as in many studies on IR (e.g., Assunção and Braido, 2007, Carletto et al., 2015; etc.), we use values of crop and farm outputs that allows for multiple crops and livestock, and further extend productivity measures to net output or profit of both crop production and total farm operations. Specifically, we used four measures of productivity: i) gross value of crop output per hectare cultivated; ii) gross value of total farm output per hectare operated; iii) net value of crop output per hectare cultivated; and iv), net value of total farm output per hectare operated.

Our study utilizes primary data collected from a cross-section of 2,000 small- and medium-scale farm operators in Nigeria as part of the Agricultural Policy Research in Africa (APRA) program, funded by the United Kingdom’s Department for International Development (UK-DFID). This new data is different from the kind of household survey data used by most previous studies on IR estimations because of the range of farm sizes covered. Farm sizes in previous nationally representative household survey data are mostly under 5 ha making such data insufficient to perform our kind of analysis due to under-representation of medium- and/or large-scale farms. In addition to the contributions mentioned above, because the new data was collected in part to test the IR hypothesis, the present study is able to account for the usual suspects (imperfect factor markets and omitted variable bias, e.g., lack of soil quality data) culpable for IR findings in the previous literature.

We find evidence of a non-linear U-shaped relationship between farm size and productivity over the range of farm sizes studied (0–40 ha). However, when restricted to only small-scale farms, we find clear evidence of an IR like many previous studies. In general, regardless of the productivity measure used, IR holds largely between zero and 22 ha, and then turns positive afterwards. When confining the medium-scale farm sample to those who “stepped-up” from small-scale farming, the turning point to a positive relationship occurred at 11 ha, showing that this type of medium-scale farms tended to exhibit a positive relationship between farm size and farm productivity at a lower threshold farm size than those who “stepped into” farming. We indeed find that medium-scale farmers have heterogeneous backgrounds that affect their productivity in ways that can be characterized according to the pathway through which such operators might have emerged. Considering our data is restricted to two representative states in Nigeria, our findings should be interpreted in this context and may not be seen as nationally representative. Nevertheless, if upheld by future studies, these findings may hold important implications for agricultural and land policy in the region.

## 2 Overview on farm size-productivity relationship

The IR literature in SSA is robust but limited mainly to smallholder farms. The literature has attributed the IR “puzzle” to a variety of factors. The most common explanations include factor market imperfections in labor where constraints on non-farm employment lead to an overuse of family labor surplus on their own farms, leading to very low shadow prices (Binswanger et al., 1995, Carter, 1984, Eswaran and Kotwal, 1986, Sen, 1966). Feder (1985) and Eswaran and Kotwal (1986) espoused a second issue, the principal-agent problem explanation whereby the cost of hired labor supervision is high relative to family labor—a moral hazard problem. Large farms tend to use hired labor more intensively than small farms, leading larger farms to be less productive per unit land than smallholder plots. In our study, we estimate farm-specific shadow wages for family labor and control for hired labor to account for labor market imperfections. On the other hand, land productivity and especially labor productivity may be higher on large-scale farms when technologies such as mechanization are utilized. Foster and Rosenzweig (2017) presented a theoretical basis for a U-shaped farm size-productivity relationship in farming based upon empirical application to India farms. The authors attributed the IR reversal among large-scale farms to economies of scale in the ability of machines to accomplish tasks at lower costs at greater operational scales.

A third class of explanations for the IR are omitted variables such as soil quality and similar unobserved heterogeneity across plots and farms (Assunção and Braido, 2007, Bhalla and Roy, 1988, Benjamin, 1995, Kimhi, 2006, Lamb, 2003). Assuming soil qualities are correlated with farm size, perhaps due to competition for farmland, omission of variables representing these qualities may bias IR estimates. However, Barrett et al. (2010) used a laboratory-tested soil quality variable and found no evidence that soil quality explains away IR results at the plot-level.^3^ Assunção and Braido (2007) investigated omitted variable bias using household fixed effects and plot level-seasonality effects and found that the IR remains intact. Controlling for land quality reveals that the IR still generally holds albeit with slightly weakened magnitude (Assunção and Braido, 2007, Benjamin, 1995, Carter, 1984). On the contrary, Bhalla and Roy (1988) used Indian data and find that controlling for farm-level soil quality eliminates the IR. However, they did not control for labor inputs and imperfections in labor markets in their study.

Fourth, smallholder risk aversion could lead to IR (Barrett, 1996, Srinivasan, 1972). For instance, land market imperfections and lack of insurance markets may actively push smallholders (usually net buyers of staple food) to invest additional labor on their farms as a mitigating factor against buying food at a more expensive price later in the market. The reverse may be the case for medium to large-scale farmers who are usually net sellers. Hence, studies that do not control for labor input may conclude that smallholders are more productive due to a correlation between farm size and risk averse behavior in the presence of multiple market failures.

Lastly, measurement errors either in self-reported plot size (Carletto et al., 2013, Carletto et al., 2015, Lamb, 2003) or output (Desiere and Jolliffe, 2018, Gourlay et al., 2019) have recently been examined in the IR literature. Studies by Carletto et al., 2013, Carletto et al., 2015 used self-reported (SR) farm size and compared farm size-productivity estimates with GPS plot measurements. Similarly, Dillon et al. (2019) compared SR farm size with GPS and the FAO recommended compass-and-rope (CR) plot measures. These studies found that small-scale farms were more likely to overstate farm size and larger farms likely to under-report farm size, but IR holds regardless of the farm area measurement approach (SR, GPS, or CR); largely supporting that previous IR findings are not statistical artifacts. In fact, Carletto et al. (2013) finds that GPS measure of farm size strengthened evidence in support of IR. However, more recent studies have suggested systematic measurement errors, particularly self-reported output or yield may be key drivers of IR findings (Desiere and Jolliffe, 2018, Gourlay et al., 2019). Both studies utilize crop-cuts to determine yield and find that IR is an artifact of measurement error, particularly, self-reported yield. Again, these studies are largely limited to samples of small-scale farms and evaluated productivity in terms of yield of a single crop.

Whereas most studies have found or upheld the existence of IR hypothesis in SSA (e.g., Obasi, 2007, Carletto et al., 2013, Ali and Deininger, 2015, Dillon et al., 2019, Julien et al., 2019), some find a U-shaped relationship between farm size and productivity (Carter and Wiebe, 1990, Kimhi, 2006, Muyanga and Jayne, 2019). For instance, Muyanga and Jayne (2019) found IR between farm size and crop productivity among small-scale farms (<3 ha) in Kenya, but the IR gives way for a U-shaped relationship when medium-scale farms were included in their sample. Similarly, Kimhi (2006) examined a relationship between maize productivity and plot size in Zambia and found a U-shaped relationship with IR dominating up to 3 ha (about 86% of their sample), but constant and increasing returns to scales beyond the 3-hectare threshold. There remains a dearth of evidence from SSA about the relationship between farm size and farm productivity when a wider range of farm sizes is considered and how the various explanations for the IR as discussed above may influence the robustness of such findings.

## 3 Data, sampling design and variables used

### 3.1 Data

**Fig. 1:**
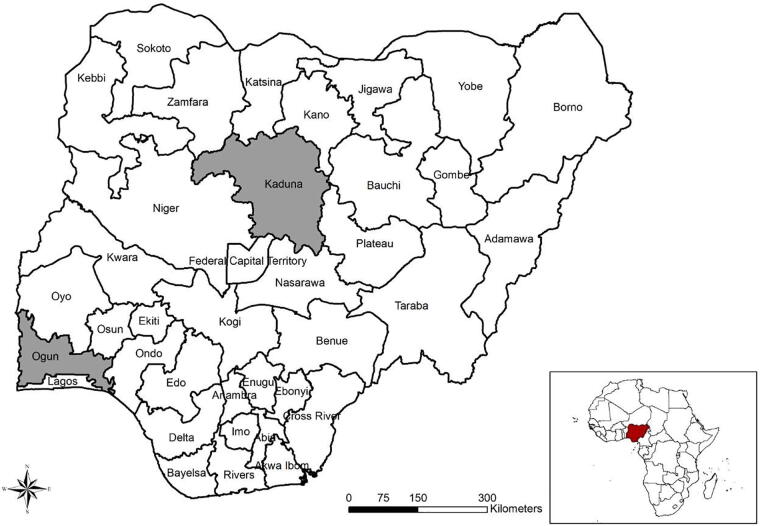
Map of Nigeria showing states with Ogun and Kaduna states highlighted.

This study uses primary cross-sectional data collected from a survey of about 2,000 farm owners/operators for farm sizes ranging up to 40 ha in Ogun and Kaduna States in Nigeria (see Fig. 1 for map of study location). The data was collected using a structured questionnaire designed to capture socio-economic information on households managing or operating these farms, agricultural inputs used, crops cultivated, animals raised, output, and marketing information. Because most farm households cultivate more than one plot, we aggregate such plots to household level. We dropped 1.8% of cultivated plots that households had not yet harvested for the agricultural season covered by the survey interviews, for which output values are missing. There is no correlation between farm size categories and the share of unharvested plots.

Collecting primary data was necessary for this study because most available farm household survey datasets in SSA tend to have very limited numbers of medium-scale farms to make inferential conclusions about them.^4^ Moreover, because urban-based households appear to constitute an unknown but potentially sizeable proportion of new investment in commercialized medium-scale farms, existing nationally-representative farm surveys may increasingly omit an important and growing segment of the population of medium-scale farms (Jayne et al., 2016). For the above reasons, a comprehensive listing and sampling of small-scale and medium-scale farm households in the study areas were conducted.

### 3.2 Sampling design

We employed a multi-stage stratified sampling strategy starting with a listing of the population of households controlling and/or operating a farm of 5 ha and above in the study area. Initial decision to compile a list of farms larger than 5 ha was to ensure a selection of representative medium/large-scale farms in this study. Unlike medium-scale farms, small-scale farms were ubiquitous and can be readily listed near medium-scale farms.

Administratively, Nigeria is divided into 36 states and the Federal Capital Territory (FCT). For this study, Ogun and Kaduna states in southern and northern Nigeria (Fig. 1), respectively, were purposively selected because both states have made significant strides in providing necessary policy environment for medium to large-scale commercial agriculture development. For example, Kaduna state was one of the five pilot states (Cross River, Enugu, Kaduna, Kano and Lagos) where the World Bank supported the implementation of the Commercial Agriculture Development Program (CADP) between 2009 and 2017. Additional reason for choosing Ogun state is its proximity to Lagos, the largest commercial center in Nigeria. Furthermore, in the past decade, the Ogun state government has adopted policies and strategies that highly favor the development of commercial agriculture.^5^ Because of this purposive selection of states, our study findings are certainly not representative of all of rural Nigeria, though the two states selected are among many where national or state-level commercial agricultural development programs have been implemented.

Nevertheless, the climatic, vegetational and rural livelihood characteristics of both states are a fair representation of the socio-economic and biophysical conditions under which over 75 percent of the Nigerian population dwells. On one hand, Kaduna state in northern Nigeria experiences a tropical continental climate with two distinct seasonal climates—dry (November-April) and rainy (May-October) seasons. The average annual rainfall, humidity and temperature are 1,272.5 mm, 56.64% and 25.14˚C, respectively. The vegetation cover is woodland and tall grass savanna. Agriculture is the main stay of Kaduna’s economy with about 80 per cent of the people actively engaged in farming. Major cash and food crops cultivated include cotton, groundnut, tobacco, maize, beans, guinea corn, millet, ginger, and rice. Moreover, it is also a trade center and a major transportation hub with rail and road access for the surrounding agricultural areas.

On the other hand, Ogun state in southern Nigeria has a humid tropical climate with distinct wet (March-September) and dry (October-February) seasons. The average annual rainfall, humidity and temperature are about 1,270 mm, 84.7% and 28°C, respectively. The vegetation cover ranges from freshwater swamps in mangrove forests to rainforest and woody guinea savanna. Major crops cultivated in the state include maize, cassava, cocoa, yam/cocoyam, oil palm, cashew, plantain, banana, citrus, mango, watermelon, vegetables, ginger, garlic, pepper. Ogun state has a high concentration of industrial estates and is a major manufacturing hub in Nigeria.

Following the selection of states, a second stage in the listing exercise included systematic selection of three Local Government Areas (LGAs) in each state.^6^ Typically, each state in Nigeria has three (3) senatorial districts to represent them at the National Assembly (The Senate). These senatorial districts are an important feature along which infrastructural investments, among other resources, are allocated. Hence, we purposively sampled one LGA per senatorial district based on land size and medium-scale farmer concentrations. The selected LGAs are Kachia, Chikun, and Soba in Kaduna South, Central, and North senatorial districts, respectively;^7^ and Ijebu East, Imeko–Afon, and Obafemi Owondo LGAs in Ogun East, West, and Central senatorial districts, respectively. The final stage of the listing exercise entailed listing of all households controlling (e.g., owned, rented-in, borrowed, etc.) or operating farms five hectares and above within the selected LGAs. The exercise was implemented by a team of 40 trained enumerators.^8^ LGAs consist of wards (administrative units within LGAs), and each ward contains several communities, which may be villages or towns.

To ensure representation of all wards in each sampled LGA, all wards were ranked based on medium-scale farm concentrations in the ward according to the listings and LGA population information, and then stratified into terciles in Kaduna, yielding three relatively equal groups: high concentration, medium concentration, and low concentration of medium-scale farms. For Ogun state with relatively smaller number of medium-scale farms, wards were stratified into quartiles, yielding four relatively equal groups: high concentration, medium–high concentration, medium–low concentration, and low concentration of medium/large farms. Subsequently, there were two levels of random selection: ward-level and farm-level. For each selected LGA, a ward was selected randomly from each of the three terciles in Kaduna state and four quartiles in Ogun State. Thus, nine wards, three from each LGA, were selected in Kaduna while in Ogun twelve wards, four from each LGA, were selected. Three wards per LGA were randomly selected in Kaduna and four wards per LGA in Ogun State. At the farm-level, in each ward, we employed a random sampling with probability proportional to size to ensure proper representation of the population of medium-scale farms operational in each LGA (see Appendix Table A1 for breakdown of samples from both states). Furthermore, our sampling design accounted for the two farming systems (smallholder farms and medium-scale farms) in the LGAs. Small-scale farmers in the same wards selected for the sampling of medium-scale farmers were also listed, followed by proportional random sampling.

Overall, about 1,000 farm households—500 small-scale and 500 medium-scale farm households—were surveyed in each state, yielding a total sample of about 2,000 respondents. Table 1 reports sample sizes and descriptive statistics used in the analysis for four (4) categories of farms: small-scale (operated farm sizes <5 ha), and three categories of medium-scale farms: 5–10, 10–20 and 20–40 ha of operated farmland. To be representative of both small- and medium-scale farm populations at the ward level, design weights proportional to size were derived at the APRA HQ by the Institute of Development Studies (IDS), Sussex. The proportion of stepped-up farmer operators in the 5–10, 10–20, and 20–40-hectare farm size categories are 53%, 40%, and 25%, respectively, suggesting that the proportion of farmers stepping up into medium-scale farming from small-scale farming reduces as farm size increases. Overall, roughly half (48.5%) of medium-scale farmers in our sample stepped-up from small-scale farming and only about 1 in 20 small-scale farm operators ever scaled-up into medium-scale farming.

**Table 1:** Descriptive statistics for variables by farm sizes and categories.

	Small-scale			Medium-scale								Full Sample
	(ha < 5)			(5 ≤ ha < 10)			(10 ≤ ha < 20)			(20 ≤ ha < 40)		
	Mean	SD		Mean	SD		Mean	SD		Mean	SD	Mean
	(1)	(2)		(3)	(4)		(5)	(6)		(7)	(8)	(9)
*A. Dependent variables*												
Gross value of crop output/ha cultivated*	327	257		298	259		306	402		277	207	323
Gross value of farm operated output/ha*	372	335		307	262		308	401		280	208	363
Net value of crop output/ha cultivated*	242	249		204	261		229	398		221	217	237
Net value of farm operated output/ha*	282	323		210	264		231	398		223	218	272
*B. Farm household characteristics*												
Age of household head (years)	43.62	13.52		45.76	12.06		50.17	12.55		49.85	13.82	44.09
Adult equivalent	5.13	2.69		5.88	2.80		6.28	2.78		7.22	3.24	5.26
=1 if female-headed household	0.06	0.24		0.04	0.18		0.01	0.12		0.01	0.11	0.06
Years of education of HH head	6.85	5.24		7.49	5.33		7.36	5.28		7.54	5.01	6.94
*C. Assets*												
=1 if HH has a radio	0.81	0.39		0.86	0.35		0.85	0.36		0.87	0.34	0.82
=1 if HH has a TV	0.29	0.45		0.40	0.49		0.50	0.50		0.51	0.50	0.31
=1 if HH has a mobile phone	0.94	0.24		0.96	0.20		0.97	0.16		0.96	0.19	0.94
=1 if HH has a motorcycle	0.65	0.48		0.67	0.47		0.78	0.42		0.81	0.39	0.66
=1 if HH has a car	0.05	0.23		0.11	0.31		0.16	0.37		0.36	0.48	0.07
=1 if HH has a water pump	0.03	0.16		0.07	0.25		0.10	0.30		0.14	0.35	0.03
=1 if HH has plow	0.14	0.35		0.18	0.38		0.11	0.31		0.13	0.34	0.15
==1 if HH has a sprayer	0.44	0.50		0.56	0.50		0.68	0.47		0.82	0.39	0.47
*D. Self-assessment of soil qualities*												
=1 if sandy soil	0.03	0.18		0.16	0.37		0.13	0.34		0.11	0.32	0.05
=1 if clay soil	0.04	0.20		0.07	0.26		0.08	0.26		0.03	0.18	0.05
=1 if loamy soil	0.97	0.17		0.91	0.28		0.95	0.22		0.95	0.22	0.96
=1 if stony soil	0.02	0.13		0.04	0.19		0.05	0.22		0.04	0.20	0.02
=1 if forest soil	0.00	0.06		0.00	0.06		0.00	0.06		0.01	0.12	0.00
= 1 if good	0.91	0.28		0.96	0.20		0.96	0.19		0.98	0.15	0.92
=1 if fair	0.13	0.33		0.12	0.32		0.11	0.32		0.06	0.23	0.12
=1 if poor+	0.01	0.08		0.01	0.09		0.03	0.16		0.00	0.00	0.01
*E. Production and input use practices*												
Farm size (ha)	2.40	1.09		6.13	1.37		12.84	2.67		24.72	5.93	3.30
Total landholding (ha)	3.81	35.30		10.58	41.73		17.55	15.94		31.12	17.39	5.17
Ratio operated farm size/total landholdings⋄ (%)	88.10	22.61		83.99	24.41		87.30	21.91		88.32	21.64	87.66
Total cost of crop production/ha planted*	85.15	67.68		93.10	86.99		76.62	75.85		54.75	86.86	85.40
Family labor days/ha	14.00	20.83		7.86	25.22		7.20	24.74		2.98	4.48	13.08
Hired labor days/ha	6.60	9.07		8.38	27.52		12.11	50.72		7.33	20.70	6.96
Estimated family labor cost/ha*	0.83	0.20		0.72	0.02		0.70	0.04		0.68	0.02	0.81
Hired labor cost/ha*	44.16	47.78		48.32	75.59		40.54	52.37		26.24	30.44	44.32
Fertilizer quantity (kg/ha)	136	106.27		127	90.84		95	88.28		69	71.54	133
Fertilizer cost (NGN/ha)*	61.50	87.12		25.45	26.32		9.82	9.29		4.93	6.00	55.77
Fertilizer price/kg (NGN)	118.69	105.58		144.54	133.24		121.06	113.94		116.60	124.31	121.32
No. of ag. related trainings attended by HH members	5.48	5.24		2.12	1.74		0.00	0.00		0.00	0.00	5.43
=1 if HH has access to market	0.44	0.50		0.31	0.46		0.29	0.45		0.17	0.38	0.42
=1 if HH has access to extension agents	0.12	0.33		0.13	0.33		0.18	0.39		0.09	0.28	0.12
=1 if HH has access to agro-input dealer	0.32	0.47		0.27	0.44		0.20	0.40		0.17	0.38	0.31
=1 if stepped-up to medium-scale farming (%)	0.00	0.00		0.53	0.50		0.40	0.49		0.26	0.44	0.07
=1 if Ogun	0.31	0.46		0.33	0.47		0.50	0.50		0.48	0.50	0.32
=1 if Kaduna	0.69	0.46		0.67	0.47	0.85	0.50	0.50		0.52	0.50	0.68
Sample size (N)	1,103			674			234			68		2079

Notes: Farm operated includes crop cultivation and animal holding operations.

* Values reported in ‘000 naira (‘000 NGN). Exchange rate is $1 = 306 Naira as at time of survey.

⋄ This ratio was computed at household level and mean results presented here.

+ Some households cultivated more than one plot and may report different soil quality on different plots. Thus, summing the share of households reporting of soil qualities (poor, fair or good) may exceed 100%.

### 3.3 Dependent and explanatory variables

For dependent variables, we computed four measures of agricultural productivity: i) the gross value of crop output per hectare cultivated, ii) the gross value of total farm output per hectare operated, iii) the net value of crop output per hectare cultivated, and iv) the net value of total farm output per hectare operated. The main difference between crop and farm output is that the latter includes income from animal products.^9^ However, there is strong correlation between these measures of crop and overall farm income,^10^ and as will be reported below, the two sets of net productivity results are highly consistent.

The net values of crop and operated farm outputs were computed as the gross values less the total cost including input costs, hired labor cost, and shadow wage imputed for family labor. Carter and Wiebe (1990) find that IR is reversed when profit measures (after deducting the cost of family labor) were examined. Hence, comparing gross and net values per hectare as computed in this study will allow us to examine whether input costs, including the cost of family labor, affect our conclusions about the relationship between farm size and productivity. Because family labor valuation may differ from observed local agricultural wage rates, we conduct robustness checks by estimating family labor in two ways: (i) estimation of a shadow wage to value unpaid household labor, following Skoufias, 1994, Abdulai and Regmi, 2000; and (ii) valuing family labor at the observed local agricultural wage rate.

The main explanatory variables of interest are the self-reported area of land cultivated or operated by farms and its squared term. The use of self-reported values as against GPS-measured value may be rightfully questioned but recent studies have demonstrated that the use of self-reported farm areas rather than GPS-measured areas does not explain away the IR (Carletto et al., 2013, Carletto et al., 2015, Dillon et al., 2019). Other explanatory variables included are demographic information of the farming household; assets including farm equipment; proxy for agro-ecology, self-reported soil type (sandy, clay, loamy, stony or forest) and quality (good, fair or poor); categories of crops cultivated—grains, legumes, roots and tubers, fruits and vegetables, and cash crops; inputs, including fertilizer use and labor (hired and family). In addition, information such as access to market and/or input dealers, access to and use of extension services were derived from asking survey respondents whether they have access to the services. Lastly, a binary indicator variable to capture whether a farm operator stepped up to medium-scale farming from prior small-scale operations (=1) and zero (0) if a farmer stepped into medium-scale farming without prior small-scale farming engagement. Previous studies examining the characteristics of medium-scale farmers find two distinct entry pathways, each with distinct socio-demographic attributes (Jayne et al., 2019).

## 4 Estimation strategy

Following previous studies (e.g. Assunção and Braido, 2007, Barrett et al., 2010, Muyanga and Jayne, 2019), we estimate a typical model for testing the existence of IR as specified in equation (1).^11^

(1) $$Y_{i}=\beta_{o}+\beta_{1}A_{i}+\beta_{2}A_{i}^{2}+\beta_{3}X_{i}+\beta_{4}S_{i}+\beta_{5}A_{i}*S_{i}+\varepsilon_{i}$$

Let $Y_{i}$ be the outcome variables (i.e., self-reported measures of productivity) for household i. The main explanatory variables of interest are the self-reported farm size/area cultivated or operated in hectares ($A_{i}$) and its square ($A_{i}^{2}$). The quadratic term is to determine shape of any curvature and turning point, if any, in the estimated function. Let ($X_{i}$) represent a vector of controls including household characteristics, family and hired labor to account for imperfect factor markets, fertilizer input use, and crop category cultivated (these are binary indicator variables for each of the crop categories mentioned earlier). Other elements in the vector $X_{i}$ are self-assessed soil types and qualities, household assets, access to agricultural extension and output market, and state fixed effect. Also included in Eq. (1) is a dummy variable $S_{i}$ to account for the two distinct modes of entry into medium-scale farming as described earlier. We suspect households who were engaged in small-scale farming prior to stepping-up to medium-scale farming may have more farm management experience or knowledge of agro-ecological and micro-climate of farming areas, than those primarily involved in non-farm jobs who later acquired land for diversification into farming or who started farming after having retired from non-farm work. This dummy variable is also interacted with the farm size variable to examine potential differences in productivity between stepped-up farm operators and others as farm size increases.

The $\beta_{s}$ (Greek beta) are parameters to be estimated where $\beta_{1}$ and $\beta_{2}$ are the main parameters of interest, $\beta_{3}$ is a vector of parameters including household and farm characteristics, $\beta_{4}$ estimates productivity for stepped-up farm operators relative to other farmers, $\beta_{5}$ captures impact of any incremental productivity attributed to stepping up as farm size increases, and $\varepsilon_{i}$ is the error term. If $\beta_{1}$ is negative and $\beta_{2}$ is positive from Eq. (1), the relationship between farm size and productivity is U-shaped (convex), confirming the existence of IR up to a certain threshold. If, however, $\beta_{1}$ is positive and $\beta_{2}$ is negative, the relationship is bell-shaped (concave), suggesting a direct relationship exists between farm size and productivity up to a certain threshold before declining. An F-test on the quadratic farm size parameter ($\beta_{2}$) determines the shape of the curve and potential turning points. The process of estimating the turning points follows directly from differential calculus. Consider the quadratic equation $y=ax^{2}+bx+c$, then the turning point is determined from the first order differentiation as $\frac{\mathrm{dy}}{\mathrm{dx}}=2ax+b=0$, where x=-b/2a is the turning point. The differential calculus approach also applies to $\beta_{4}$ and $\beta_{5}$ to examine incremental productivity among medium-scale farmers depending on their mode of entry.

For each productivity measure, we first estimated a parsimonious model with farm size and exogenous household variables, including market and agricultural extension access, in vector ($X_{i}$). Subsequently, we included potentially endogenous variables such as labor and fertilizer use, crop types cultivated, soil qualities, and decision to step up farming from small-scale to medium-scale farming. Including labor ensures that we control for market imperfection in labor use as alluded to in previous IR studies. Lastly, we controlled for household assets to account for wealth, farm equipment, access to both output and input markets, and crop composition dummies to control for potential correlation between farm size and the type of crops grown (Dorward, 1999).

Overall, we estimated different variants of Eq. (1) as Models I-IV. In Model I, the sample size is restricted to small-scale farms (<5ha) only. We estimated a separate model for small-scale farms because most of the previous studies on IR in SSA have been within this range of farms. Thus, we can examine if the usual findings of IR among smallholders is sustained in the Nigerian context. Models II and III include samples from both small- and medium-scale farms but restricts the medium-scale sub-sample to only *stepped-in* or *stepped-up* farmers, respectively. The rationale for this is to examine how the relationship between farm size and productivity differ based on the type of medium-scale farm household. According to previous research, those *stepping-in* to medium-scale farming in some cases lacked prior farming experience or it was a secondary endeavor for them, whereas the *stepped-up* farmers were primarily engaged as small-scale farming before ‘stepping-up’ to medium-scale farming. A difference between estimated Models II and III would suggest presence of heterogeneity in productivity of medium-scale farm operators, depending on the pathways through which such medium-scale farms have emerged. Finally, contingent on the results of a Chow test to determine whether it is appropriate to pool the sub-samples of stepped-up and stepped-in medium-scale farms, we estimate Eq. (1) using the full sample (Model IV), including a dummy variable for stepped-up farmers and an interaction term between this dummy and the linear farm size variable to test for differences in productivity between the two types of medium-scale farms. All regressions, including multivariate and univariate estimations for descriptive statistics, used population weights that are inversely proportional to the probability of being sampled at the ward level.

## 5 Results and discussion

### 5.1 Descriptive statistics

To provide an at-a-glance understanding of the distribution of farm sizes in our sample and how their characteristics may vary across size categories, we present descriptive statistics across four different farm size categories. These categorizations, shown in Table 1, were not used for regression purposes but rather to show how basic characteristics vary across the four categories of farms. The dependent variables (measures of productivity in panel A) are on average highest for the small-scale farms, lowest for farms between 5 and 10 ha, rising again for farms between 10 and 20 ha and then declining again beyond 20 ha. In addition, the net values (gross values less total cost of production from labor and input use) of crop and total farm output per hectare suggest that on average, small-scale farms are also most productive with productivity decreasing in the 5 to 10-hectare medium-scale farms, then increased again for farms beyond 10 ha. The reason for the decreasing and then increasing net crop or farm productivity values seems connected with the medium-scale farms having a much lower crop production cost per hectare once farm size is larger than 10 ha (see Panel E). Total cost of production per hectare is highest for farms within the 5–10-hectare range explaining the dip in net productivity in this range. These findings, in general, may suggest a U-shaped curve in productivity as farm size increases.

Panel B shows differences across farm size categories operated in terms of household head’s age, family size (adult equivalent), gender of household head, and years of education. On average, operators of medium-scale farms are older and have larger household sizes (adult equivalent). Farming households with over 10 ha have about two adults more than small-scale households on average. In addition, smallholder farms are significantly more likely to be headed by a female relative to medium-scale farms, and years of education of household heads is slightly higher for medium-scale farm operators. Some medium-scale farm operators may be urban-based professionals, retirees or influential rural dwellers as found in a recent study of medium/large farms in sub-Saharan Africa (Jayne et al., 2016).

On household assets (Panel C), on average, about 82% of farm operators have access to a radio regardless of farm size operated. Mobile phone ownership is nearly universal across farm size at between 94% and 97% ownership. However, significant differences are observed in ownership of high-value assets such as motorcycles, cars, water pumps, and mechanical sprayers. Across the categories, the proportion of farm operators owning these high-value assets is low among small-scale farmers and increases as farm size increases with farms larger than 20 ha having highest ownerships.

Panel D shows self-reported soil type and quality characteristics. Given the potential correlation between soil quality, fertilizer use, and farm size, results suggest that self-assessment of soil types and quality are largely the same across farm size categories. Most farms (96%) identified their soil type as loamy while a similar share assessed their soil quality as good.

In Panel E, the proportion of land cultivated or operated is positively correlated with total landholdings across all farm size categories, averaging about 88% across farm size categories. This contrasts with findings from Kenya, where the proportion of land operated is inversely proportional to total landholdings among medium-scale farms (Muyanga & Jayne, 2019). This contrast may be attributed to land security or land tenure systems where Kenya has more freeholdings and registered lands as against customary tenure or leaseholds in Nigeria. The fear of losing parts of acquired lands through revocation of use rights by community or government, if left unused, is palpable in Nigeria. On the other hand, the total cost of production per hectare declines with farm size, consistent with findings from Kenya (Muyanga & Jayne, 2019), except for the 5 to 10-hectare farms. The lower unit cost among bigger medium-scale farms may represent economies of scale in production. In addition, consistent with previous studies identifying imperfect labor market as a contributor to IR results (e.g., Carter, 1984, Sen, 1966, etc.), we find evidence that family labor is most intensively used on small-scale farms. Family labor days per hectare significantly decreases as farm size increases. Family labor per hectare on small-scale farms is almost five times higher than on medium-scale farms over 20 ha. However, hired labor days per hectare increases with farm size and is significantly higher than family labor per hectare across all farm size categories except among smallholders. The estimated family shadow wage for family labor is very low relative to hired labor, supporting evidence of labor market imperfections suggested in previous studies.

On input use, we find fertilizer use per hectare to be high at 133 kg/ha, on average. This is consistent with Sheahan and Barrett (2017), who found that fertilizer use among Nigerian farmers was 128 kg/ha. Further scrutiny reveals that fertilizer is used more intensively in Kaduna state in northern Nigeria than Ogun state in the south. This finding of more fertilizer use in northern part of Nigeria has been documented in the literature (e.g., Liverpool-Tasie, 2014). Fertilizer use and cost per hectare both decrease significantly as farm size increases.

Lastly, although equal numbers of small-scale and medium-scale farms were sampled in Ogun and Kaduna states in Southern and Northern Nigeria, respectively, applying sample weights show that 69% of small-scale farms are from Kaduna states. Similarly, 67% of samples between 5 and 10 ha are from Kaduna state. The samples are evenly distributed beyond the 10-hectare threshold.

Considering one of our study’s objectives is to examine the IR hypothesis for heterogeneity based on modes of entry into medium-scale farming, Table 2 shows the same descriptive characteristics, but only for medium-scale farm operators disaggregated by mode of entry (*stepped-up* vs. *stepped-in*). Overall, findings suggest that across all productivity measures, on average, *stepped-up* farmers (with prior farming engagement as smallholders) are more productive than their *stepped-in* counterparts. Furthermore, the stepped-up farm household heads are older, with less formal education, and more likely to be female. The two groups are relatively similar for asset wealth, input use, and soil qualities.

**Table 2:** Descriptive statistics for variables by medium-scale farmer types (5–70 ha).

	Stepped-up			Stepped-in	
Variables	Mean	SD		Mean	SD
*A. Dependent variables*					
Gross value of crop output/ha cultivated (‘000 NGN)	307	264		291	318
Gross value of farm operated output/ha (‘000 NGN)	313	264		300	322
Net value of crop output/ha cultivated (‘000 NGN)	214	267		210	318
Net value of farm operated output/ha (‘000 NGN)	218	267		216	320
*B. Farm household characteristics*					
Age of household head (years)	48.16	12.21		45.87	12.51
Adult equivalent	6.37	2.95		5.77	2.72
=1 if female-headed household	0.04	0.19		0.02	0.14
Years of education of HH head	7.06	5.23		7.83	5.35
*C. Assets*					
=1 if HH has a radio	0.87	0.33		0.83	0.37
=1 if HH has a TV	0.42	0.49		0.43	0.50
=1 if HH has a mobile phone	0.96	0.19		0.96	0.20
=1 if HH has a motorcycle	0.69	0.46		0.72	0.45
=1 if HH has a car	0.15	0.36		0.12	0.32
=1 if HH has a water pump	0.08	0.27		0.08	0.27
=1 if HH has plow	0.25	0.43		0.08	0.27
==1 if HH has a sprayer	0.65	0.48		0.57	0.50
*D. Self-assessment of soil qualities*					
=1 if sandy soil	0.18	0.39		0.12	0.32
=1 if clay soil	0.08	0.28		0.06	0.23
=1 if loamy soil	0.93	0.25		0.91	0.28
=1 if stony soil	0.04	0.20		0.04	0.19
=1 if forest soil	0.00	0.07		0.00	0.06
= 1 if good	0.97	0.18		0.96	0.21
=1 if fair	0.10	0.30		0.13	0.33
=1 if poor	0.01	0.11		0.01	0.11
*E. Production and input use practices*					
Farm size (ha)	7.74	4.04		9.77	6.44
Total landholding (ha)	11.93	27.05		14.80	43.56
Ratio operated farm size/total landholdings (%)	81.90	25.01		87.54	22.40
Total cost of crop production/ha planted (‘000 NGN)	92.95	87.58		81.18	82.13
Family labor days/ha	7.12	15.07		7.69	30.39
Hired labor days/ha	10.58	37.46		7.77	29.58
Estimated family labor cost/ha (‘000 NGN)	0.72	0.03		0.71	0.03
Hired labor cost/ha (‘000 NGN)	50.28	72.94		40.34	64.52
Fertilizer quantity (kg/ha)	119.06	99.15		112.78	82.47
Fertilizer cost (kg/ha)	22.58	27.23		19.02	20.50
Fertilizer price/kg (NGN)	119.98	108.27		121.15	108.20
No. of ag. related trainings attended by HH members	2.40	1.67		0.00	0.00
=1 if HH has access to market	0.39	0.49		0.20	0.40
=1 if HH has access to extension agents	0.16	0.37		0.11	0.32
=1 if HH has access to agro-input dealer	0.28	0.45		0.21	0.41
=1 if Ogun	0.43	0.50		0.34	0.47
=1 if Kaduna	0.57	0.50		0.66	0.47
Sample size (N)	462			522	

An important difference between the two groups is their farm size. Because *stepped-up* farmers were defined as small-scale farmers who expanded their operations, most farm only slightly more land than the threshold of 5 ha of operated land; none farm more than 25 ha. By contrast, medium-scale farmers who *stepped into* farming by investing in land are more likely to own relatively large tracts; and all farms over 25 ha are of this type.

Total production costs per hectare and hired labor days and cost per hectare is higher for stepped-up farmers. Family labor days and estimated shadow wage per hectare are similar across both categories. Moreover, fertilizer use and fertilizer cost per hectare are slightly higher for the stepped-up group of farmers. Lastly, on average, the stepped-up farmers attended at least two agricultural-related training sessions (extension programs) in the past year while the stepped-in farmers reportedly attended none. These may suggest that *stepped-up* farm operators may be more productive than their *stepped-in* counterparts.

### 5.2 Refined productivity distribution

**Fig. 2:**
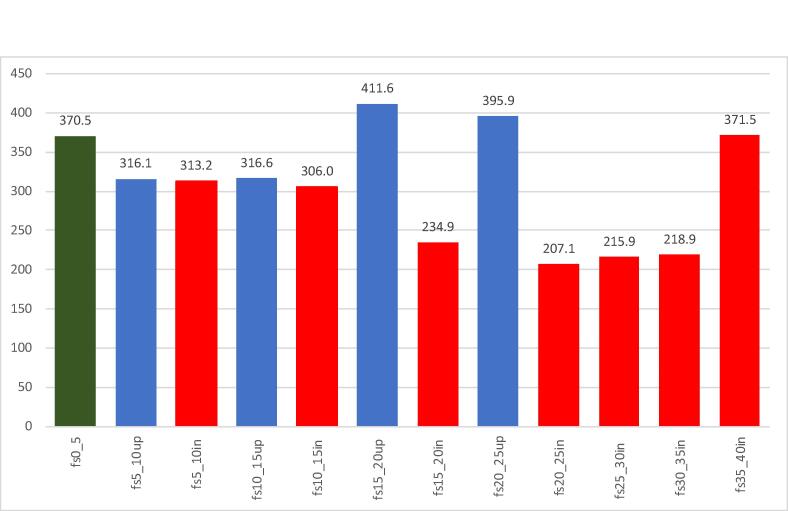
Mean distribution of gross farm output/ha (‘000) by farm size and emergence pathway (stepped-up or stepped-in) into medium-scale farming. Distribution is at 5-hectare intervals. Note: fs*_**up represents farm size ranging from * to ** for a stepped-up operator. fs*_**in represents farm size ranging from * to ** for a stepped-in operator.

Fig. 2 shows a more refined distribution of gross farm output per hectare by farm size, using 5-hectare intervals from zero to 40 ha, and distinguishing between *stepped-up* and *stepped-in* medium-scale farms. Results reveal that small-scale (0–5 ha) farmers are more productive than most other categories. Although productivity subsequently declined for 5–10 ha and 10–15 ha farms, the stepped-up farmers were slightly more productive in this range. Between 15 and 20 ha and 20–25 ha, *stepped-up* farmers are significantly more productive than *stepped-in* farmers. As there are no stepped-up farmers beyond the 25-hectare threshold, it is not possible to compare productivity levels among stepped-up and stepped-in farms beyond this range. Yet it is clear that the land productivity measures of *stepped-in* farmers within the 25–30 ha and 30–35 ha are quite low compared to the other categories, which may indicate a lack of farming experience. The exception is for stepped-in farmers in the 35–40 ha category, where productivity measures are relatively high. This might be due to these farmers’ greater use of machinery, but this is speculative because we unfortunately lack data on machinery use.

**Table:** 

Farm size	f5-10	f10-15	f15-20	f20-25	f25-30	f30-35	f35-40
**Step-up (N)**	325	73	24	19	0	0	0
**Step-in (N)**	326	99	37	27	9	5	8

### 5.3 Multivariate regression results

Table 3 reports the estimated relationship between farm size and the gross value of farm output per hectare operated, while Table 4 reports the relationship between farm size and the net value farm output per hectare operated. We later present and discuss the relationships between farm size and gross crop output per hectare cultivated—one of the conventional measures of inverse relationship—as well as farm size and net crop output per hectare, under the *robustness check* sub-section below. For all result tables, we first show estimated results for the small-scale farms (<5ha), Model I, in the first two columns. The small-scale productivity estimates were separated to enable comparison with prior findings from the literature in SSA on similar farm sizes. In the subsequent columns, we present Models II-IV showing estimates from stepped-in operators, stepped-up operators, and full-sample, respectively.^12^ In addition, for all regression models, we initially regressed output per hectare on farm size, its quadratic term and all household characteristics in a parsimonious regression. Thereafter, we added full set of control variables to account for potential confounders such as farm and soil characteristics and input use. Doing this should help examine robustness of IR productivity estimates as input decision variables are added to the model. Lastly, the quadratic terms included in each model, together with the linear term, is used to determine if there is a non-linear relationship between farm size and our measures of productivity; if the joint null hypothesis that the coefficients on the linear and squared farm size terms are rejected by an F-test, we report the turning point.

**Table 3:** Multivariate regression of gross farm output/ha operated (‘000 NGN).

	SMALL-SCALE SAMPLE (0–5 ha)			(0–5 ha) + Step-in (5–40 ha) sample			(0–5 ha) + Step-up (5–25 ha) sample			FULL SAMPLE	
										All Combined (0–40 ha)	
	Model I (1)	Model I (2)		Model II* (3)	Model II* (4)		Model III+ (5)	Model III+ (6)		Model IV⋄ (7)	Model IV⋄ (8)
Farm Size (ha)	−130.21***	−94.70**		–33.19***	–32.78***		−45.00***	−45.41***		−27.65***	−31.11***
	(45.69)	(45.51)		(7.12)	(6.96)		(11.73)	(11.03)		(5.89)	(6.68)
Squared Farm Size (ha)	17.89**	10.93		0.79***	0.75***		2.06***	2.03***		0.69***	0.69***
	(8.84)	(8.90)		(0.23)	(0.22)		(0.60)	(0.56)		(0.20)	(0.21)
Age HH head (years)	0.55	0.94		0.93	1.13		0.78	1.12		0.96	1.19
	(1.03)	(1.05)		(0.94)	(0.96)		(0.94)	(0.95)		(0.88)	(0.89)
Household size	8.63	5.34		5.86	3.06		6.98	3.50		5.49	2.84
	(5.92)	(6.07)		(5.36)	(5.43)		(5.30)	(5.41)		(4.91)	(4.97)
=1 if female headed HH	−50.12	−82.15*		−54.11	−78.63*		−45.49	−70.92*		−48.64	−69.99*
	(41.95)	(43.30)		(38.64)	(40.37)		(38.39)	(39.43)		(36.18)	(37.62)
Years of education of HH head	−2.61	−4.43*		−1.59	−3.22		−1.95	−3.34		−1.47	−2.68
	(2.52)	(2.41)		(2.38)	(2.25)		(2.38)	(2.27)		(2.24)	(2.11)
=1 if Ogun state	68.15*	−142.98*		67.90**	−135.29**		70.22**	−123.57*		69.64**	−122.97*
	(37.69)	(74.38)		(33.80)	(67.07)		(34.57)	(70.14)		(31.63)	(64.12)
Family labor days/ha		0.60			0.76**			0.57			0.61*
		(0.47)			(0.37)			(0.43)			(0.36)
Hired labor days/ha		3.43**			2.26**			1.11			1.03
		(1.71)			(0.91)			(0.88)			(0.64)
Fertilizer (kg/ha)		−0.07			−0.05			−0.05			−0.02
		(0.19)			(0.17)			(0.18)			(0.17)
Dummy variables (access to market, extensions, agro-dealer)	Yes	Yes		Yes	Yes		Yes	Yes		Yes	Yes
Dummy variables for crop categories (grains, legumes, roots & tubers, fruits & vegetables, cash crops)		Yes			Yes			Yes			Yes
HH assets (radio, TV, mobile phone, car, motorcycle)	Yes	Yes		Yes	Yes		Yes	Yes		Yes	Yes
Farm equipment (water pump, tractor, sprayer)	Yes	Yes		Yes	Yes		Yes	Yes		Yes	Yes
Soil quality & types		Yes			Yes			Yes			Yes
Dummy variables for Local Government Areas (LGAs)	Yes	Yes		Yes	Yes		Yes	Yes		Yes	Yes
=1 if HH ‘stepped up’ from small-scale											−130.81***
											(40.14)
‘Stepped up’*farm size interaction term											19.62***
											(4.76)
Constant	335.39***	373.63***		220.21***	349.57***		239.73***	280.17***		214.52***	318.55***
	(81.54)	(130.06)		(58.22)	(106.85)		(58.12)	(105.67)		(55.16)	(99.26)
Observations	1,103	1,103		1,637	1,637		1,565	1,565		2,079	2,079
R-squared	0.11	0.18		0.10	0.16		0.09	0.15		0.09	0.14
Turning point for cultivated farm size (ha)				21.0	21.8		10.9	11.2		20.0	22.5
Turning point for cultivated farm size based on stepping up (ha)											6.7

Robust standard errors in parentheses. *** p < 0.01, ** p < 0.05, * p < 0.1.

Notes: *Model II comprises small and medium-scale farmers that stepped into medium-scale farming with no prior experience as small-scale farmers (stepped-in).

+ Model III comprises small-scale and medium-scale farmers that stepped into medium-scale farming with prior experience as small-scale farmers (stepped-up).

⋄ Model IV is the full sample including small and medium-scale farmers with stepped-in and stepped-up farmers inclusive.

**Table 4:** Multivariate regression of net farm output/ha operated (‘000 NGN) with family labor valued at shadow wage.

VARIABLES	SMALL-SCALE SAMPLE (0–5 ha)			(0–5 ha) + Step-in (5–40 ha) sample			(0–5 ha) + Step-up (5–25 ha) sample			FULL SAMPLE	
										All Combined (0–40 ha)	
	Model I (1)	Model I (2)		Model II* (3)	Model II* (4)		Model III+ (5)	Model III+ (6)		Model IV^⋄^ (7)	Model IV^⋄^ (8)
Farm Size (ha)	−91.77**	−57.92		−29.27***	−26.94***		−38.08***	−34.60***		−24.98***	−24.80***
	(43.41)	(44.62)		(6.91)	(6.84)		(11.35)	(10.74)		(5.71)	(6.54)
Squared Farm Size (ha)	11.90	5.56		0.72***	0.62***		1.73***	1.59***		0.65***	0.54***
	(8.44)	(8.74)		(0.22)	(0.21)		(0.58)	(0.54)		(0.19)	(0.20)
Age HH head (years)	0.84	1.02		1.22	1.31		0.99	1.19		1.20	1.33
	(1.00)	(1.04)		(0.92)	(0.94)		(0.92)	(0.94)		(0.86)	(0.87)
Household size	7.66	3.90		5.01	1.92		6.12	2.44		4.55	1.75
	(5.59)	(5.77)		(5.05)	(5.15)		(5.00)	(5.13)		(4.63)	(4.71)
=1 if female headed HH	−46.85	−69.35		−53.44	−70.48*		−41.74	−60.89		−47.57	−62.59*
	(39.49)	(42.19)		(36.70)	(39.21)		(36.34)	(38.43)		(34.47)	(36.66)
Years of education of HH head	−3.07	−4.33*		−2.25	−3.43		−2.72	−3.68*		−2.28	−3.09
	(2.45)	(2.38)		(2.31)	(2.21)		(2.31)	(2.23)		(2.17)	(2.07)
=1 if Ogun state	85.12**	−106.50		80.71**	−106.79*		86.09**	−94.43		83.28***	−97.68
	(37.16)	(71.39)		(33.24)	(64.53)		(34.04)	(67.48)		(31.09)	(61.64)
Family labor days/ha		0.85*			0.91***			0.86**			0.84**
		(0.45)			(0.35)			(0.40)			(0.34)
Hired labor days/ha		0.06			−0.12			−0.75			−0.65
		(1.33)			(0.70)			(0.58)			(0.45)
Fertilizer (kg/ha)		−0.14			−0.12			−0.13			−0.11
		(0.19)			(0.17)			(0.18)			(0.17)
Dummy variables (access to market, extensions, agro-dealer)	Yes	Yes		Yes	Yes		Yes	Yes		Yes	Yes
Dummy variables for crop categories (grains, legumes, roots & tubers, fruits & vegetables, cash crops)		Yes			Yes			Yes			Yes
HH assets (radio, TV, mobile phone, car, motorcycle)	Yes	Yes		Yes	Yes		Yes	Yes		Yes	Yes
Farm equipment (water pump, tractor, sprayer)	Yes	Yes		Yes	Yes		Yes	Yes		Yes	Yes
Soil quality & types		Yes			Yes			Yes			Yes
Dummy variables for Local Government Areas (LGAs)	Yes	Yes		Yes	Yes		Yes	Yes		Yes	Yes
=1 if HH ‘stepped up’ from small-scale											−144.30***
											(39.12)
‘Stepped up’*farm size interaction term											19.58***
											(4.49)
Constant	205.71***	292.82**		134.17**	299.50***		152.61***	236.35**		134.18**	274.72***
	(79.44)	(128.57)		(57.16)	(104.31)		(56.95)	(103.12)		(54.15)	(96.38)
Observations	1,103	1,103		1,637	1,637		1,565	1,565		2,079	2,079
R-squared	0.11	0.16		0.10	0.15		0.09	0.14		0.09	0.14
Turning point for cultivated farm size (ha)				20.5	21.7		11.0	10.9		19.2	22.8
Turning point for cultivated farm size based on stepping up (ha)											7.4

Robust standard errors in parentheses. *** p < 0.01, ** p < 0.05, * p < 0.1.

Notes: *Model II comprises small and medium-scale farmers that stepped into medium-scale farming with no prior experience as small-scale farmers (stepped-in).

+ Model III comprises small-scale and medium-scale farmers that stepped into medium-scale farming with prior experience as small-scale farmers (stepped-up).

#### 5.3.1 Gross farm output per hectare

Column (1) in Table 3 shows evidence that within the range of farms smaller than five hectares, there is a significant inverse relationship between farm size and gross farm output per hectare. Adding further controls for soil characteristics, crop types, input use and labor in column (2) reduced the IR magnitude by 27%, indicating that not accounting for these variables for smallholder farmers may overstate the magnitude of the IR. Moreover, the quadratic term after controlling for input variables is now imprecisely estimated, suggesting no strong non-linearities over the 0–5-hectare range. These findings are generally consistent with previous literature in SSA where smallholders cultivating 5 ha or less have been the focus (Carletto et al., 2013, Carletto et al., 2015, Julien et al., 2019).

When the small-scale sample was combined with stepped-in and stepped-up medium-scale samples in Models II and III, respectively, estimates in columns (3) to (6) show that the coefficient on the linear farm size term is consistently negative while the squared term is consistently positive—a U-shaped relationship. As shown in columns (4) and (6), the addition of more control variables to account for input use and labor did not significantly change the signs or magnitude of either the linear or squared farm size variables, indicating that differences in input use do not explain the U-shaped relationship observed.

Comparing IR estimates between both modes of entry into medium-scale farming suggests existence of heterogeneity in productivity, depending on pathway of emergence. In previous IR literature, productivity of medium-scale farmers in SSA was assumed homogenous (Muyanga & Jayne, 2019). However, evidence from columns (4) and (6) suggest that stepped-up farmers with prior engagement in small-scale farming are more productive than their stepped-in counterparts without prior small-scale farming activities. For the stepped-up farmers, the turning point threshold at which IR turns to increasing return to scale is estimated at 11 ha, about half the threshold for their stepped-in counterpart at 22 ha.

Results for Model IV are not discussed here because Chow test results indicate that the coefficients of the variables in Models II and III are sufficiently different that a pooled estimation is not warranted. However, interested readers may want to evaluate these results in any case and hence we discuss them in the section on robustness checks below.

#### 5.3.2 Net farm output per hectare

Given the advantage of lower production cost that bigger farms possess over small-scale farms (see Table 1), it is important to analyze the relationship between farm size and productivity in terms of net farm output (gross farm output less total production cost including valuation of family labor) per hectare. Table 4 shows regression results of net farm output per hectare on farm size.

For small-scale farms (0–5 ha), parameter estimates reveal the presence of IR in net productivity in column (1), but with lower magnitude relative to gross productivity estimates (Table 3). The quadratic term is imprecisely estimated, suggesting no strong non-linearities over the 0–5-hectare range. When additional labor and input use decision covariates are added in column (2), the IR becomes even smaller and imprecisely estimated. These findings suggest that IR is weakened or non-existent for the net values of farm output (profit) per hectare relative to the gross output per hectare. These findings of weakened IR are generally consistent with previous literature that IR between productivity and farm size is either weakened or reversed when profit (net value) is used to measure productivity (Carter and Wiebe, 1990, Lamb, 2003). Carter and Wiebe (1990) documents that profit (similarly defined as gross value of output less total costs including family labor) increases monotonically with farm size unlike gross value of output, which was U-shaped.

Beyond the 5-hectare farms, however, we find the evidence of a U-shaped relationship again. The quadratic terms are consistently precisely estimated (Columns 3 to 6). The U-shaped relationship persists even after accounting for the use of labor and other inputs, consistent with findings in Ali and Deininger (2015) from Rwanda. Despite a weakened IR magnitude across both stepped-in and stepped-up estimates, the turning point thresholds are quite similar for both gross and net farm outputs per hectare, indicating consistency across similar productivity measures. In columns (4) to (6), we equally find evidence of heterogeneity in net farm productivity among medium-scale farmers depending mode of entry into farming. Stepped-up farmers are estimated to attain a positive turning point threshold at 10.9 ha (column (6)), twice as fast as their stepped-in counterparts at 21.7 ha.

In summary, three trends emerged across the measures of productivity above. First, there is evidence of IR between farm size and productivity within small-scale farms in Nigeria. Second, for medium-scale farms, a U-shaped relationship emerges suggesting that IR holds up to a certain turning point threshold within this farm category. This threshold consistently lies largely between 10 and 25 ha depending on productivity measure estimated or mode of entry into farming. Although Muyanga and Jayne (2019) estimated a similar U-shaped curve in a study of similar farm size (0–60 ha) in Kenya, the IR observed in Kenya was mostly restricted to smallholders and the turning point thresholds occurred at a smaller farm size than in Nigeria. Perhaps, size-productivity relationship may differ by region according to agro-ecological conditions, technology, degree of agricultural market development, and overall development status (Otsuka et al., 2016, Rada and Fuglie, 2019). Third, beyond approximately 7 ha, we find evidence that medium-scale farm operators who were small-scale farmers are more productive than other medium-scale farms run by counterparts without extensive prior farming experience. This finding, coupled with the fact that stepped-up farmers are consistently estimated to reach turning points twice as fast as their stepped-in counterparts, suggests heterogeneity within medium-scale farmers depending on mode of entry into farming. Reporting of such heterogeneity in productivity depending how the operator transitions into medium-scale farming is new in the literature and in SSA.

### 5.4 Robustness checks

#### 5.4.1 Potential endogeneity

First, we acknowledge the potential endogeneity of farm size in estimating the relationship between farm size and productivity. While farm size is endogenous, the IR studies in the literature typically use farm size as in the present study, without instrumenting for it. This is probably because the aim is to empirically examine how farm productivity varies across farm sizes, recognizing that there are potentially numerous unobserved factors correlated with farm size that are responsible for the relationship. Hence, we acknowledge that the farm size variable may be picking up on unobserved effects correlated with farm size, but this in no way invalidates our findings about the relationship between farm size and farm productivity.^13^

#### 5.4.2 Quantile regression

Like most previous IR studies, we reported parameter estimates at conditional means of productivity and not across the entire distribution of productivity. However, productivity may change within the distribution (Savastano & Scandizzo, 2017). Therefore, we estimated a quantile regression across the productivity distribution to examine if IR holds across the distribution. We find that although the relationship between farm size and land productivity is not constant over the entire productivity distribution, the signs of the estimated parameters however do not change within the distribution. The linear farm size parameter estimates consistently remain negative and significant while the quadratic term is consistently positive and significant. That is, the U-shaped relationship is maintained throughout the distribution (see Appendix Table A2).

#### 5.4.3 Gross and net crop outputs per hectare

Having presented estimates of the relationship between farm size and farm output per hectare above, we now examine the robustness of the above estimates using gross and net crop output per hectare productivity measures (removing income from animal production). Table A3 and Table A4 present these results. While the same U-shaped relationship emerges, the quadratic farm size term tends to be less precisely measured; it is statistically significant only at the 10% level in several models presented in Table A3. The turning point thresholds occurred at a higher size of farms (about 25 ha) for the stepped-in medium-scale farms, and at a lower size of farm for the stepped-up farms, at 10.4 ha. The results for the net value of crop output per hectare are very similar (Table A4). Overall, the U-shaped relationship is maintained regardless of whether productivity is measured in terms of gross or net value of output, or farm output including animal income or just crop output.

#### 5.4.4 Log-log estimates

We argue that level-level estimation is the most straightforward way to test the relationship between farm size and farm productivity. Log-log estimation, while often applied in IR studies, produces coefficient estimates of the relationship between percentage changes in farm size and percentage changes in productivity, which is technically different than the relationship between farm size and productivity. One could easily envision cases whereby increases in farm size are associated with increasing levels of productivity, but declining percentage changes in productivity (or the reverse) at the margin. For this reason, we present results from level-level models as the preferred models. Nevertheless, as a robustness check, we estimated a log–log variant of the gross farm output models in Table 3. Although few of the farm size terms approach a high level of statistical significance, the signs and magnitudes of the estimated parameters of interest are similar to the main results (Appendix Table A5).

#### 5.4.5 Sensitivity of net productivity measure to family labor valuation

Table A6 replicates the main result in Table 4 (net farm output per hectare) but with family labor valued at local agricultural (hired labor) wage rate as against household specific shadow wage in the main table. The idea is that given smallholders tend to use family labor intensively, they may appear more or less productive if the shadow wage is considerably different than hired wage. In this study, the estimated shadow price for family labor was very low relative to the local agricultural wage rate on average. However, the family labor cost is a relatively small proportion of the total cost of production compared to hired labor and other inputs such as fertilizers. For this reason, the magnitude and statistical significance of the coefficients of interest remain highly consistent regardless of whether family labor is valued at the observed local agricultural wage rate or the much lower shadow rate (Table A6).

#### 5.4.6 Estimation of pooled sample

Although Chow test suggests that a pooled estimation for the two medium-scale farm types is not warranted, we discuss the estimated results of the pooled sample (Model IV) for all productivity measures here. First, columns (7) and (8) in Table 3 show estimation results for gross farm output per hectare when all samples (0–40 ha) are combined. Results show that the IR magnitude is further reduced relative to either farm type, and the quadratic term remains positive and statistically significant. These estimates indicate a U-shaped relationship between farm size and productivity, and that IR exists up to a certain threshold beyond which farm productivity increases as farm size increases. The turning point is estimated at about 22 ha over the entire sample. As shown in Fig. 2, this U-shaped relationship appears largely driven by the stepped-up medium-scale farmers in the 15–25 ha range and the stepped-in farmers in the 35–40 ha range. The farmers in this range are more efficient with fertilizer input use and overall total cost of production is lower (Table 1). Our findings are consistent with Carter and Wiebe, 1990, Muyanga and Jayne, 2019 who earlier found a U-shaped relationship between farm output and farm size in Kenya.

On the impact of prior farming engagement on mediums-scale farm productivity, the negative parameter estimates on $\beta_{4}$ suggest that, on average, relative to all other farmers (including small-scale farmers), stepped-up farmers may be less productive in general. However, the positive estimate of the interaction term between stepped-up binary indicator variable and farm size demonstrates that prior farming engagement is positively correlated with gross farm productivity as farm size increases beyond a certain farm size. A partial derivative of the productivity function with respect to $S_{i}$ reveals that beyond 6.7 ha, the effect of stepping up is positive relative to stepped-in medium-scale and further confirms heterogeneity in productivity within medium-scale farms.

For net farm productivity in Table 4, the pooled estimations in columns (7) and (8) similarly finds that the U-shaped relationship between farm size and net farm productivity is sustained with a turning point at 22.8 ha. In addition, beyond the 7.4-hectare threshold (column 8, last row), stepped-up farmers become more productive than their stepped-in counterparts. This finding, again, confirms the existence of heterogeneity in productivity for medium-scale farms depending on mode of entry into farming.

Finally, in Table A3 the pooled sample estimates in columns (7) and (8) equally maintain the U-shaped relationship between farm size and gross crop productivity measures, with both parameters of interest precisely estimated. The turning point is estimated at 25 ha, and stepped-up farmers become distinctly more productive beyond approximately 6.2 ha. Table A4 presents estimations results of the relationship between farm size and net crop productivity when both medium-scale farm types are pooled. Results are largely identical to the pooled gross crop productivity estimates.

## 6 Conclusion and policy implications

This study examines the relationship between farm size and productivity using data from two states in Nigeria that have supported commercial agricultural investments by medium- and large-scale farms. While our results cannot be considered representative of Nigeria, it is noted that at least 15 of Nigeria’s 36 states have adopted similar programs and policies to encourage commercial agricultural investments.

The study makes several contributions to the farm size/farm productivity literature. First, to our knowledge, this study is one of only two studies to have examined the farm size/productivity relationship over a relatively wide range of farm sizes that include medium-scale farms operating up to 40 ha of land. The importance of this is twofold. First, medium-scale farms appear to be a rapidly rising segment of African agriculture in Nigeria and many other African countries (Jayne et al., 2019). Second, current debates about land tenure policy in Africa have focused on the merits of making it easier for commercial investor farmers, both national and foreign, to acquire relatively large tracts of land in areas formerly reserved for local smallholder communities (African Development Bank, 2017). One of the assertions justifying this approach is that large farms are more entrepreneurial and productive than relatively poor smallholder farmers (Collier & Dercon, 2014). The academic IR literature has often been invoked as a counterweight to these charges, pointing out that small farms tend to be more productive than larger ones. However, these assertions are unfortunately on shaky ground because hardly any IR studies from Africa use samples containing more than a few farms cultivating more than 10 or even 5 ha. So, while the policy questions being asked are crucially important, in our view, greater interrogation of the relevance of the IR literature is necessary to inform contemporary land policy debates in Sub-Saharan Africa. Our study is an attempt to overcome this lacuna by developing a sampling frame capable of evaluating land productivity differences between small-scale (0–5 ha) and medium-scale (5–40 ha) farms.

The study’s second contribution builds on observations from prior studies indicating that medium-scale farms in Africa are a highly heterogenous group. Many relatively large farms are owned by investors primarily engaged in non-farm employment, who live and work outside the area where they purchased their farms. Some may rely on relatives to work the farm while they visit on weekends. Other medium-scale farms are derived from successful small-scale farmers who re-invested their proceeds to acquire more land and make farm capital investments. We hypothesized that it would be important to differentiate between these two types of medium-scale farms when examining the relationship between farm size and productivity. It may be possible, for example, that one type of medium-scale farm is relatively productive while another is not, which may in turn hold important implications for land and rural development policies.

A third contribution of this study is that it utilizes several measures of land productivity that go beyond simple and potentially misleading measure such as the yield of a given staple crop or even the value of crop output per hectare. The importance of livestock products in African agriculture warrant the inclusion of whole farm operations, not just crops, and this study is one of the few to have included animal output in measures of farm productivity. Moreover, we look at the robustness of results based on gross farm output vs. net farm output measures of productivity, the latter deducting the costs of family labor, hired labor and other cash inputs.

Three main findings are highlighted here. First, consistent with previous literature in SSA region, we find evidence that the inverse relationship (IR) exists within small-scale (<5ha) agriculture in Nigeria. Second, when the sample is expanded to include farms up to 40 ha of operated land, a U-shaped relationship emerges between farm size and productivity – a conclusion that is highly robust to choice of productivity indicator, differences in model specification, estimator, and valuation of family labor. Third, we find systematic differences in the productivity of medium-scale farms according to the owner’s mode of entry into farming. Medium-scale farmers with prior farming activities as small-scale farmers were more productive than counterparts who stepped into medium-scale farming with little or no prior farming activities, at least up to 25 ha.

Considering the paucity of empirical evidence showing IR hypothesis beyond smallholder farms, the above findings may hold important policy implications for agricultural policy development in SSA if upheld by future studies in SSA. First, our findings of a U-shaped farm size-productivity relationship suggests the pursuit of policies that emphasize a need to highlight support to both small-scale farms as well as farms greater 15 ha or so. Because roughly half of the current medium-scale farms were formerly small-scale farmers who expanded their operations, it is possible that appropriate support to small-scale farm households may enable the productivity growth of smallholders as well as the development of a new commercialized class of medium-scale farms that step-up from small-scale operations. In this way, development support for smallholder farms may simultaneously promote the development of a wide scale of farming operations.

Second, because medium-scale farmers with prior small-scale farming experience appear more productive between 5 and 25 ha and attain IR turning point thresholds at faster rates (at about half the threshold) than those who stepped into medium-scale farming without prior small-scale farming activities, policies encouraging smallholder access to larger agricultural land may not only improve the living standards of smallholder communities but also enhance agricultural productivity. Given about 1 in 20 small-scale farms in Nigeria have actually scaled up so far, this ratio could rise appreciably with proper support. However, population growth in rural areas presents a challenge to consolidate agricultural lands as many smallholders acquire lands through family inheritance and land subdivision. Thus, any policy to enhance agricultural land consolidation among smallholders to enhance agricultural productivity should focus on structural transformation creating jobs to absorb the burgeoning rural population in Nigeria. Nevertheless, there is also some evidence that farmers who stepped into medium-scale farming without prior small-scale farming show relatively high level of productivity beyond 35 ha.

Moreover, we stress that comparisons of farm productivity are but one criterion of importance to African governments and societies when considering land policy decisions. Other relevant development criteria include which scale of farming generates the strongest multiplier effects on rural non-farm employment and broader economy-wide transformation. A stylized fact from Asia’s experience show that agricultural growth linkages with the rest of the economy are stronger with a unimodal (smallholder-led) rather than bimodal farm structure (Johnston & Kilby, 1975; Mellor, 1995), although these and other authors also found certain synergies resulting from a mix of large and commercialized small farms in the same vicinity. General equilibrium effects resulting from agricultural growth under alternative agrarian structures may potentially outweigh differences in crop output per hectare due to scale of operation. For these reasons, we regard the findings of this study as an important but incomplete contribution towards understanding the complex developmental effects of alternative farm structures.

Lastly, our sample is limited to two states in Nigeria (one in the north and another in the south) that have created an enabling environment for medium-scale agriculture to thrive. Our findings therefore should be interpreted in this context and may not be seen as nationally representative. We recommend more studies considering diverse regions, multiple agro-ecologies, and different levels of economic growth or development to generate more evidence for generalization.

## Author contributions

OO conducted all the analysis and did most of the writing. TJ did some of the writing, checking and confirming to ensure analysis was done correctly. MM did some data collection and reviewed earlier draft. AA supervised the data collection and reviewed manuscript at various stages. SOL reviewed earlier draft of the manuscript. TA reviewed earlier draft of the manuscript. All authors were involved in idea formulation or evolution of overarching research goals.

## Declaration of Competing Interest

The authors declare that they have no known competing financial interests or personal relationships that could have appeared to influence the work reported in this paper.

## Footnotes

1 In other regions such as Latin America, the farm size-productivity relationship has been more extensively examined over a wide range of farm sizes (e.g., Rada et al., 2019;Helfand and Taylor, 2020).

2 Sources of productivity differences between these two types of medium-scale farms include farming or farm management experience, knowledge about the local agro-ecology and microclimate, and local social capital leading to ability to get good quality or easily accessible land, labor, and understanding local farm input/output market dynamics.

3 An important element of land quality that Barrett et al. and the present study did not include is land topography. Whether land is contiguous, relatively flat or hilly, rocky or interspersed with trees or tree stumps can influence its suitability for large-scale farming through mechanization. Many small farms are less suitable for mechanization for these reasons. When small plots are combined together into a large farm, it may not be as productive as a comparably sized farm of only one field. We thank Keith Fuglie for raising this point.

4 For example, the 2010/11 Tanzania Living Standards Measurement Survey (LSMS) contains 11 farms cultivating between 20 and 50 ha, and only one farm between 50 and 100 ha. In the Uganda LSMS, there are 12 farms between 20 and 50 ha and none over 50 ha. The Malawi 2010/11 LSMS contains one farm observation between 10 and 20 ha, 1 farm between 20 and 50 ha, and zero farms over 50 ha. The recently released Nigeria 2018/19 LSMS data has only 10 observations between 10 and 60 ha. These surveys do not contain enough sample size to draw meaningful conclusions about farms over 20 ha (Christiaensen and Demery, 2018, p. 10).

5 Refer to https://ogunagric.com/investor-relations/

6 States in Nigeria are administratively divided into Local Government Areas (LGAs), which themselves are further divided into wards comprising many communities, villages or towns.

7 While Birnin Gwari LGA was the largest in Kaduna Central, it could not be included in this study owing to insecurity concerns. Consequently, Chikun—the next largest LGA in Kaduna Central—was selected.

8 The listing protocol used by enumerators is available upon request.

9 The livestock income variable includes 4 components: the value of (i) live animal sales; (ii) slaughtered livestock sold as meat; (iii) sales of livestock products such as eggs and milk; and (iv) animal products consumed by the household.

10 Pairwise correlations between crop and farm output is about 86%.

11 Other studies (e.g., Carletto et al., 2013; Desiere and Jolliffe, 2018; Dillon et al., 2019) have used log–log variant of estimating Eq. (1) in the form of elasticities. Although we estimated a direct level-level relationship between productivity and farm size, as a robustness check, we equally estimated a log–log variant of the model above.

12 To ensure that data from the different categories of medium-scale farm operators can be pooled, we performed a Chow Test to determine whether the samples from Model II and III can be pooled. We reject the null hypothesis that the coefficients in the model are similar for the two sub-samples of medium-scale farms, suggesting that a pooled estimation is not warranted. However, we still report these estimates later as a robustness check on the main results.

13 We note that a recent *meta*-review of the IR literature (Delvaux, Riesgo, and Paloma, 2020) does not even discuss endogeneity because in a full data world in which all relevant variables in the production function were controlled for, there would be no statistical relationship between farm size and productivity. It is the omitted variables correlated with farm size that empirically drive the farm size/productivity relationship.
