# Supplementary material for: A revisit of farm size and productivity: Empirical evidence from a wide range of farm sizes in Nigeria
Source: World Dev. 2021 Oct;146:105592. doi: 10.1016/j.worlddev.2021.105592 (PMC8350315; doi:10.1016/j.worlddev.2021.105592)
Supplement: Supplementary data 1 [file mmc1.docx]

APPENDIX

**Table A.1a: Medium and Smallholder Farm Samples in Ogun State**

|  |  | **Medium/large Farms** | |  | **Smallholder Farms** | |
| --- | --- | --- | --- | --- | --- | --- |
| *Local Government Authority [LGA]* | *Ward* | *Population* | *Sample* |  | *Population* | *Sample* |
| Obafemi Owondo | Owode | 169 | 71 |  | 264 | 16 |
|  | Alapako | 109 | 46 |  | 1,154 | 72 |
|  | Oba | 64 | 27 |  | 330 | 20 |
|  | Obafemi | 57 | 24 |  | 939 | 58 |
|  |  | 399 | 167 |  | 2,687 | 167 |
| Ijebu East | Owu | 181 | 76 |  | 309 | 41 |
|  | Itele | 157 | 66 |  | 254 | 33 |
|  | Imobi | 33 | 14 |  | 340 | 45 |
|  | Ikija | 27 | 11 |  | 368 | 48 |
|  |  | 398 | 167 |  | 1,271 | 167 |
| Imeko – Afon | Imeko | 275 | 102 |  | 635 | 63 |
|  | Atapele | 94 | 35 |  | 544 | 54 |
|  | Obada | 54 | 20 |  | 286 | 28 |
|  | Agberiodo | 28 | 10 |  | 219 | 22 |
|  |  | 451 | 167 |  | 1,684 | 167 |
|  | Total | 1,248 | 500 |  | 5,642 | 500 |

**Table A.1b: Medium and Smallholder Farm Samples in Kaduna State**

|  |  | **Medium/large Farms** | |  | **Smallholder Farms** | |
| --- | --- | --- | --- | --- | --- | --- |
| *Local Government Authority [LGA]* | *Ward* | *Population* | *Sample* |  | *Population* | *Sample* |
| Chikun | Rido | 228 | 93 |  | 1,127 | 65 |
|  | Kuriga | 94 | 38 |  | 501 | 29 |
|  | Kunai | 87 | 35 |  | 1,266 | 73 |
|  |  | 409 | 167 |  | 2893 | 167 |
| Kachia | Agunu | 390 | 87 |  | 3301 | 105 |
|  | Gidan Tagwai | 230 | 51 |  | 1202 | 38 |
|  | Bishini | 129 | 29 |  | 727 | 23 |
|  |  | 749 | 167 |  | 5230 | 167 |
| Soba | Gami Gira | 346 | 111 |  | 1694 | 59 |
|  | Dan Wata | 116 | 37 |  | 1488 | 52 |
|  | Garu | 60 | 19 |  | 1593 | 56 |
|  |  | 522 | 167 |  | 4775 | 167 |
|  | Total | 1,680 | 500 |  | 12,899 | 500 |

Table A.2: QUANTILE REGRESSION OF GROSS FARM OUTPUT/HA OPERATED (‘000 NGN)

| VARIABLES | Q10 | Q25 | Q50 | Q75 | Q90 |
| --- | --- | --- | --- | --- | --- |
|  | (1) | (2) | (3) | (4) | (5) |
| Farm Size (ha) | -10.37*** | -12.06*** | -10.64*** | -14.32*** | -22.90*** |
|  | (1.57) | (2.15) | (3.27) | (3.19) | (6.94) |
| Squared Farm Size (ha) | 0.29*** | 0.29*** | 0.26** | 0.37*** | 0.55*** |
|  | (0.06) | (0.07) | (0.12) | (0.14) | (0.20) |
| Age HH head (years) | 0.17 | 0.41* | 0.28 | 0.31 | 2.88** |
|  | (0.23) | (0.23) | (0.38) | (0.54) | (1.43) |
| Household size | 0.40 | 0.86 | 2.39 | 4.99* | 0.58 |
|  | (1.47) | (1.30) | (2.14) | (2.97) | (4.75) |
| =1 if female headed HH | -24.48 | -27.46** | -17.34 | -10.05 | -36.76 |
|  | (16.83) | (12.93) | (24.06) | (23.17) | (78.22) |
| Years of education of HH head | 0.04 | 0.21 | 0.48 | 0.94 | 3.88 |
|  | (0.51) | (0.67) | (1.11) | (1.74) | (2.56) |
| =1 if Ogun state | 13.68 | -1.69 | 16.43 | 12.39 | -30.06 |
|  | (17.96) | (17.06) | (18.11) | (43.93) | (79.31) |
| Family labor days/ha | -0.03 | -0.18 | 0.01 | 0.56* | 0.48 |
|  | (0.12) | (0.22) | (0.42) | (0.34) | (0.54) |
| Hired labor days/ha | -0.03 | 0.11 | 0.23 | 1.73 | 2.18 |
|  | (0.17) | (0.20) | (0.56) | (1.11) | (1.48) |
| Fertilizer (kg/ha) | 0.20*** | 0.09 | 0.07 | 0.14 | 0.20 |
|  | (0.06) | (0.07) | (0.06) | (0.12) | (0.23) |
| =1 if grains | 42.96*** | 54.65*** | 8.66 | -32.35** | 8.14 |
|  | (10.16) | (15.00) | (22.98) | (15.98) | (60.07) |
| =1 if legumes | -3.79 | -4.14 | -1.95 | -7.36 | -22.41 |
|  | (7.37) | (8.08) | (12.62) | (16.92) | (38.60) |
| =1 if roots & tubers | 14.44** | 26.03*** | 38.85*** | 102.33*** | 240.99*** |
|  | (6.14) | (8.70) | (14.64) | (31.48) | (35.07) |
| =1 if fruits & vegetables | 11.98 | 16.79** | 16.72 | 46.91* | 73.57* |
|  | (8.53) | (7.09) | (13.46) | (24.78) | (39.17) |
| =1 if cash crop | 32.44** | 41.06*** | 21.53 | 35.95 | 70.19 |
|  | (13.84) | (10.05) | (15.37) | (28.11) | (51.12) |
| Access dummies (market, extensions, agro-dealer) | Yes | Yes | Yes | Yes | Yes |
| Soil quality & types | Yes | Yes | Yes | Yes | Yes |
| Household assets (radio, TV, mobile phone, car, motorcycle) | Yes | Yes | Yes | Yes | Yes |
| Farm equipment (water pump, tractor, sprayer) | Yes | Yes | Yes | Yes | Yes |
| =1 if HH ‘stepped up’ from small-scale | -28.12* | -33.72 | -44.84 | -5.76 | -76.86 |
|  | (15.78) | (21.10) | (29.15) | (59.91) | (69.72) |
| ‘Stepped up’*farm size | 3.46** | 5.45* | 7.04** | 9.73 | 25.82*** |
|  | (1.73) | (3.05) | (2.74) | (6.57) | (7.04) |
| Constant | 8.01 | 62.18** | 161.45*** | 291.89*** | 319.32*** |
|  | (31.72) | (28.68) | (54.14) | (69.82) | (107.01) |
| Observations | 2,079 | 2,079 | 2,079 | 2,079 | 2,079 |
| Pseudo R-squared | 0.06 | 0.05 | 0.05 | 0.07 | 0.13 |
| Turning point for cultivated farm size (ha) | 17.6 | 21.1 | 20.7 | 19.4 | 20.7 |

Table A.3: MULTIVARIATE REGRESSION OF GROSS CROP OUTPUT/HA CULTIVATED (‘000 NGN)

|  | SMALL-SCALE SAMPLE | |  | (0-5ha) + | | | | (0-5ha) + | | | | FULL SAMPLE | | |
| --- | --- | --- | --- | --- | --- | --- | --- | --- | --- | --- | --- | --- | --- | --- |
|  | (0-5 ha) | |  | Step-in (5-40 ha) sample | | |  | | Step-up (5-25 ha) sample | |  | | All Combined (0-40 ha) | |
| VARIABLES | Model I  (1) | Model I  (2) |  | Model II^*^  (3) | | Model II^*^ (4) |  | | Model III^+^ (5) | Model III^+^ (6) |  | | Model IV^◊^ (7) | Model IV^◊^  (8) |
| Farm Size (ha) | -105.55*** | -77.43** |  | -16.41*** | -17.94*** | |  | | -19.67** | -22.63*** |  | | -12.70*** | -17.24*** |
|  | (35.91) | (35.62) |  | (5.35) | (5.27) | |  | | (8.46) | (7.94) |  | | (4.35) | (5.11) |
| Squared Farm Size (ha) | 18.08** | 12.04* |  | 0.34* | 0.36** | |  | | 1.00** | 1.08*** |  | | 0.28* | 0.34** |
|  | (7.11) | (7.04) |  | (0.18) | (0.17) | |  | | (0.45) | (0.42) |  | | (0.15) | (0.17) |
| Age HH head (years) | 1.01 | 1.37* |  | 1.34* | 1.56** | |  | | 1.17 | 1.50** |  | | 1.34* | 1.58** |
|  | (0.83) | (0.82) |  | (0.76) | (0.75) | |  | | (0.76) | (0.75) |  | | (0.71) | (0.70) |
| Adult equivalent | 0.60 | -2.16 |  | -0.73 | -3.25 | |  | | 0.04 | -2.97 |  | | -0.52 | -2.92 |
|  | (4.11) | (4.04) |  | (3.82) | (3.68) | |  | | (3.72) | (3.64) |  | | (3.50) | (3.38) |
| =1 if female headed HH | -20.22 | -54.48 |  | -23.19 | -51.38 | |  | | -17.57 | -45.94 |  | | -19.50 | -44.71 |
|  | (34.83) | (35.28) |  | (33.11) | (33.79) | |  | | (32.65) | (32.94) |  | | (31.40) | (31.83) |
| Years of education of HH head | 1.48 | -0.03 |  | 2.07 | 0.70 | |  | | 1.82 | 0.67 |  | | 1.99 | 0.97 |
|  | (1.87) | (1.70) |  | (1.79) | (1.62) | |  | | (1.78) | (1.62) |  | | (1.68) | (1.52) |
| =1 if Ogun state | 70.77** | -139.67** |  | 66.37** | -138.73** | |  | | 70.68** | -126.54** |  | | 66.73*** | -129.03** |
|  | (30.90) | (60.04) |  | (27.66) | (53.86) | |  | | (28.13) | (56.00) |  | | (25.69) | (51.23) |
| Family labor days/ha |  | 0.39 |  |  | | 0.52* |  | |  | 0.37 |  | |  | 0.40 |
|  |  | (0.39) |  |  | | (0.31) |  | |  | (0.37) |  | |  | (0.32) |
| Hired labor days/ha |  | 2.67** |  |  | | 1.76*** |  | |  | 0.76 |  | |  | 0.74 |
|  |  | (1.18) |  |  | | (0.64) |  | |  | (0.69) |  | |  | (0.50) |
| Fertilizer (kg/ha) |  | 0.03 |  |  | | 0.04 |  | |  | 0.04 |  | |  | 0.06 |
|  |  | (0.13) |  |  | | (0.12) |  | |  | (0.12) |  | |  | (0.11) |
| Dummy variables (access to market, extensions, agro-dealer) | Yes | Yes |  | Yes | | Yes |  | | Yes | Yes |  | | Yes | Yes |
| Dummy variables for crop categories (grains, legumes, roots & tubers, fruits & vegetables, cash crops) |  | Yes |  |  | | Yes |  | |  | Yes |  | |  | Yes |
| HH assets (radio, TV, mobile phone, car, motorcycle) | Yes | Yes |  | Yes | | Yes |  | | Yes | Yes |  | | Yes | Yes |
| Farm equipment (water pump, tractor, sprayer) | Yes | Yes |  | Yes | | Yes |  | | Yes | Yes |  | | Yes | Yes |
| Soil quality & types |  | Yes |  |  | | Yes |  | |  | Yes |  | |  | Yes |
| Dummy variables for Local Government Areas (LGAs) | Yes | Yes |  | Yes | | Yes |  | | Yes | Yes |  | | Yes | Yes |
| =1 if HH ‘stepped up’ from small-scale |  |  |  |  | |  |  | |  |  |  | |  | -85.17** |
| Dummy variables for Local Government Areas (LGAs) | Yes | Yes |  | Yes | | Yes |  | | Yes | Yes |  | | Yes | Yes |
| ‘Stepped up’*farm size interaction term |  |  |  |  | |  |  | |  |  |  | |  | 13.79*** |
|  |  |  |  |  | |  |  | |  |  |  | |  | (4.15) |
| Constant | 267.98*** | 245.10*** |  | 161.57*** | | 231.75*** |  | | 172.57*** | 161.27** |  | | 158.75*** | 210.65*** |
|  | (60.77) | (89.72) |  | (42.70) | | (77.12) |  | | (42.95) | (71.29) |  | | (39.91) | (70.87) |
| Observations | 1,103 | 1,103 |  | 1,637 | | 1,637 |  | | 1,565 | 1,565 |  | | 2,079 | 2,079 |
| R-squared | 0.08 | 0.19 |  | 0.07 | | 0.16 |  | | 0.06 | 0.16 |  | | 0.06 | 0.15 |
| Turning point for cultivated farm size (ha) | 2.9 | 3.2 |  | 24.0 | | 24.7 |  | | 9.8 | 10.4 |  | | 22.5 | 25.4 |
| Turning point for cultivated farm size based on stepping up (ha) |  |  |  |  | |  |  | |  |  |  | |  | 6.2 |

Robust standard errors in parentheses. *** p<0.01, ** p<0.05, * p<0.1

Notes: ^*^Model II comprises small and medium-scale farmers that stepped into medium-scale farming with no prior experience as small-scale farmers (stepped-in)

^+^Model III comprises small-scale and medium-scale farmers that stepped into medium-scale farming with prior experience as small-scale farmers (stepped-up)

^◊^Model IV is the full sample including small and medium-scale farmers with stepped-in and stepped-up farmers inclusive.

Table A.4: MULTIVARIATE REGRESSION OF NET CROP OUTPUT/HA CULTIVATED (‘000 NGN)

|  | SMALL-SCALE SAMPLE | |  | (0-5ha) + | | | (0-5ha) + | | | | FULL SAMPLE | | |
| --- | --- | --- | --- | --- | --- | --- | --- | --- | --- | --- | --- | --- | --- |
|  | (0-5 ha) | |  | Step-in (5-40 ha) sample | |  | | Step-up (5-25 ha) sample | |  | | All Combined (0-40 ha) | |
| VARIABLES | Model I  (1) | Model I  (2) |  | Model II^*^  (3) | Model II^*^ (4) |  | | Model III^+^ (5) | Model III^+^ (6) |  | | Model IV^◊^ (7) | Model IV^◊^  (8) |
| Farm Size (ha) | -73.53** | -47.47 |  | -13.58*** | -13.08** |  | | -14.53* | -13.62* |  | | -11.00*** | -11.88** |
|  | (33.70) | (34.73) |  | (5.11) | (5.10) |  | | (8.01) | (7.53) |  | | (4.15) | (4.94) |
| Squared Farm Size (ha) | 13.02* | 7.69 |  | 0.30* | 0.26 |  | | 0.75* | 0.72* |  | | 0.27* | 0.22 |
|  | (6.77) | (6.92) |  | (0.17) | (0.17) |  | | (0.42) | (0.40) |  | | (0.14) | (0.16) |
| Age HH head (years) | 1.25 | 1.39* |  | 1.58** | 1.67** |  | | 1.34* | 1.52** |  | | 1.53** | 1.66** |
|  | (0.81) | (0.81) |  | (0.74) | (0.74) |  | | (0.74) | (0.74) |  | | (0.69) | (0.69) |
| Household size | 0.32 | -3.05 |  | -1.01 | -3.94 |  | | -0.24 | -3.60 |  | | -0.94 | -3.60 |
|  | (3.86) | (3.83) |  | (3.58) | (3.47) |  | | (3.49) | (3.43) |  | | (3.29) | (3.18) |
| =1 if female headed HH | -16.74 | -41.46 |  | -22.27 | -42.78 |  | | -13.69 | -35.57 |  | | -18.25 | -36.84 |
|  | (32.45) | (34.26) |  | (31.20) | (32.69) |  | | (30.69) | (32.00) |  | | (29.72) | (30.92) |
| Years of education of HH head | 1.07 | -0.03 |  | 1.48 | 0.44 |  | | 1.11 | 0.28 |  | | 1.25 | 0.52 |
|  | (1.81) | (1.70) |  | (1.72) | (1.60) |  | | (1.72) | (1.60) |  | | (1.62) | (1.50) |
| =1 if Ogun state | 83.84*** | -105.85* |  | 77.07*** | -112.39** |  | | 82.95*** | -99.39* |  | | 78.18*** | -105.45** |
|  | (30.47) | (57.29) |  | (27.19) | (51.57) |  | | (27.70) | (53.53) |  | | (25.26) | (48.98) |
| Family labor days/ha |  | 0.64* |  |  | 0.68** |  | |  | 0.67** |  | |  | 0.63** |
|  |  | (0.36) |  |  | (0.29) |  | |  | (0.32) |  | |  | (0.28) |
| Hired labor days/ha |  | -0.51 |  |  | -0.52 |  | |  | -1.02** |  | |  | -0.88*** |
|  |  | (0.90) |  |  | (0.50) |  | |  | (0.42) |  | |  | (0.33) |
| Fertilizer (kg/ha) |  | -0.05 |  |  | -0.04 |  | |  | -0.05 |  | |  | -0.03 |
|  |  | (0.13) |  |  | (0.12) |  | |  | (0.12) |  | |  | (0.11) |
| Dummy variables (access to market, extensions, agro-dealer) | Yes | Yes |  | Yes | Yes |  | | Yes | Yes |  | | Yes | Yes |
| Dummy variables for crop categories (grains, legumes, roots & tubers, fruits & vegetables, cash crops) |  | Yes |  |  | Yes |  | |  | Yes |  | |  | Yes |
| HH assets (radio, TV, mobile phone, car, motorcycle) | Yes | Yes |  | Yes | Yes |  | | Yes | Yes |  | | Yes | Yes |
| Farm equipment (water pump, tractor, sprayer) | Yes | Yes |  | Yes | Yes |  | | Yes | Yes |  | | Yes | Yes |
| Soil quality & types |  | Yes |  |  | Yes |  | |  | Yes |  | |  | Yes |
| Dummy variables for Local Government Areas (LGAs) | Yes | Yes |  | Yes | Yes |  | | Yes | Yes |  | | Yes | Yes |
| =1 if HH ‘stepped up’ from small-scale |  |  |  |  |  |  | |  |  |  | |  | -101.02*** |
|  |  |  |  |  |  |  | |  |  |  | |  | (33.02) |
| ‘Stepped up’*farm size interaction term |  |  |  |  |  |  | |  |  |  | |  | 14.05*** |
|  |  |  |  |  |  |  | |  |  |  | |  | (3.88) |
| Constant | 148.50** | 172.80* |  | 80.19* | 185.37** |  | | 90.20** | 120.28* |  | | 82.85** | 170.55** |
|  | (58.68) | (88.90) |  | (41.07) | (75.30) |  | | (41.29) | (69.44) |  | | (38.40) | (68.74) |
| Observations | 1,103 | 1,103 |  | 1,637 | 1,637 |  | | 1,565 | 1,565 |  | | 2,079 | 2,079 |
| R-squared | 0.09 | 0.17 |  | 0.08 | 0.15 |  | | 0.08 | 0.16 |  | | 0.07 | 0.14 |
| Turning point for cultivated farm size (ha) |  |  |  | 22.8 | 25.4 |  | | 9.7 | 9.4 |  | | 20.6 |  |
| Turning point for cultivated farm size based on stepping up (ha) |  |  |  |  |  |  | |  |  |  | |  | 7.2 |

Robust standard errors in parentheses. *** p<0.01, ** p<0.05, * p<0.1

Notes: ^*^Model II comprises small and medium-scale farmers that stepped into medium-scale farming with no prior experience as small-scale farmers (stepped-in)

^+^Model III comprises small-scale and medium-scale farmers that stepped into medium-scale farming with prior experience as small-scale farmers (stepped-up)

^◊^Model IV is the full sample including small and medium-scale farmers with stepped-in and stepped-up farmers inclusive.

Table A.5: LOG-LOG REGRESSION OF GROSS FARM OUTPUT/HA OPERATED (‘000 NGN)

|  | SMALL-SCALE SAMPLE | |  | (0-5ha) + | | (0-5ha) + | | |  | | FULL SAMPLE | |
| --- | --- | --- | --- | --- | --- | --- | --- | --- | --- | --- | --- | --- |
|  | (0-5 ha) | |  | Stepped-in (5-40 ha) sample | |  | Stepped-up (5-25 ha) sample | |  | All Combined (0-40 ha) | | |
| VARIABLES | Model I  (1) | Model I  (2) |  | Model II^*^  (3) | Model II^*^ (4) |  | Model III^+^ (5) | Model III^+^ (6) |  | Model IV^◊^ (7) | | Model IV^◊^  (8) |
| Log farm size | -0.93 | -0.95 |  | -0.96 | -0.69 |  | -0.82 | -0.60 |  | -0.91 | | -0.83 |
|  | (0.65) | (1.21) |  | (0.63) | (1.11) |  | (0.65) | (1.15) |  | (0.63) | | (1.09) |
| Log square farm size | 0.34 | 0.29 |  | 0.34 | 0.19 |  | 0.29 | 0.15 |  | 0.33 | | 0.26 |
|  | (0.32) | (0.58) |  | (0.31) | (0.54) |  | (0.32) | (0.56) |  | (0.31) | | (0.53) |
| Log HH head’s age | -0.00 | 0.10 |  | 0.02 | 0.13 |  | 0.01 | 0.13 |  | 0.03 | | 0.14 |
|  | (0.09) | (0.12) |  | (0.09) | (0.11) |  | (0.09) | (0.11) |  | (0.08) | | (0.10) |
| Log adult equivalent | 0.11* | 0.05 |  | 0.09* | 0.04 |  | 0.09* | 0.03 |  | 0.09* | | 0.04 |
|  | (0.06) | (0.07) |  | (0.05) | (0.07) |  | (0.05) | (0.07) |  | (0.05) | | (0.06) |
| =1 if female headed HH | -0.04 | -0.18 |  | -0.05 | -0.17 |  | -0.04 | -0.17 |  | -0.05 | | -0.16 |
|  | (0.10) | (0.13) |  | (0.10) | (0.12) |  | (0.10) | (0.12) |  | (0.09) | | (0.11) |
| Log head’s years of education | -0.02 | -0.03 |  | -0.02 | -0.03 |  | -0.02 | -0.03 |  | -0.02 | | -0.03 |
|  | (0.02) | (0.02) |  | (0.02) | (0.02) |  | (0.02) | (0.02) |  | (0.02) | | (0.02) |
| =1 if Ogun state | 0.19** | -0.42** |  | 0.17** | -0.40** |  | 0.18** | -0.36* |  | 0.17** | | -0.35** |
|  | (0.08) | (0.21) |  | (0.08) | (0.18) |  | (0.08) | (0.19) |  | (0.07) | | (0.17) |
| Log family labor days/ha |  | 0.02 |  |  | 0.01 |  |  | 0.02 |  |  | | 0.01 |
|  |  | (0.02) |  |  | (0.02) |  |  | (0.02) |  |  | | (0.02) |
| Log hired labor days/ha |  | 0.02 |  |  | 0.03 |  |  | 0.03 |  |  | | 0.03 |
|  |  | (0.03) |  |  | (0.03) |  |  | (0.03) |  |  | | (0.02) |
| Log fertilizer (kg/ha) |  | 0.06 |  |  | 0.10** |  |  | 0.05 |  |  | | 0.09** |
|  |  | (0.06) |  |  | (0.05) |  |  | (0.05) |  |  | | (0.04) |
| Dummy variables (access to market, extensions, agro-dealer) | Yes | Yes |  | Yes | Yes |  | Yes | Yes |  | Yes | | Yes |
| Dummy variables for crop categories (grains, legumes, roots & tubers, fruits & vegetables, cash crops) |  | Yes |  |  | Yes |  |  | Yes |  |  | | Yes |
| HH assets (radio, TV, mobile phone, car, motorcycle) | Yes | Yes |  | Yes | Yes |  | Yes | Yes |  | Yes | | Yes |
| Farm equipment (water pump, tractor, sprayer) | Yes | Yes |  | Yes | Yes |  | Yes | Yes |  | Yes | | Yes |
| Soil quality & types |  | Yes |  |  | Yes |  |  | Yes |  |  | | Yes |
| Dummy variables for Local Government Areas (LGAs) | Yes | Yes |  | Yes | Yes |  | Yes | Yes |  | Yes | | Yes |
| =1 if HH ‘stepped up’ from small-scale |  |  |  |  |  |  |  |  |  |  | | -0.79** |
|  |  |  |  |  |  |  |  |  |  |  | | (0.37) |
| Log ‘stepped up’*farm size interaction term |  |  |  |  |  |  |  |  |  |  | | 0.32** |
|  |  |  |  |  |  |  |  |  |  |  | | (0.14) |
| Constant | 13.29*** | 12.83*** |  | 13.25*** | 12.54*** |  | 13.20*** | 12.50*** |  | 13.19*** | | 12.53*** |
|  | (0.48) | (0.95) |  | (0.45) | (0.83) |  | (0.46) | (0.86) |  | (0.44) | | (0.78) |
| Observations | 1,103 | 593 |  | 1,637 | 984 |  | 1,565 | 883 |  | 2,079 | | 1,261 |
| R-squared | 0.11 | 0.28 |  | 0.11 | 0.27 |  | 0.10 | 0.26 |  | 0.10 | | 0.25 |

Robust standard errors in parentheses. *** p<0.01, ** p<0.05, * p<0.1

Notes: ^*^Model II comprises small and medium-scale farmers that stepped into medium-scale farming with no prior experience as small-scale farmers (stepped-in)

^+^Model III comprises small-scale and medium-scale farmers that stepped into medium-scale farming with prior experience as small-scale farmers (stepped-up)

^◊^Model IV is the full sample including small and medium-scale farmers with stepped-in and stepped-up farmers inclusive.

Table A.6: MULTIVARIATE REGRESSION OF NET FARM OUTPUT/HA OPERATED (‘000 NGN) WITH FAMILY LABOR VALUED AT HIRED WAGE

|  | SMALL-SCALE SAMPLE | |  | (0-5ha) + | |  | (0-5ha) + | |  | FULL SAMPLE | |
| --- | --- | --- | --- | --- | --- | --- | --- | --- | --- | --- | --- |
|  | (0-5 ha) | |  | Stepped-in (5-40 ha) sample | |  | Stepped-up (5-25 ha) sample | |  | All Combined (0-40 ha) | |
| VARIABLES | Model I  (1) | Model I  (2) |  | Model II^*^  (3) | Model II^*^ (4) |  | Model III^+^ (5) | Model III^+^ (6) |  | Model IV^◊^ (7) | Model IV^◊^  (8) |
| Farm Size (ha) | -91.77** | -57.92 |  | -29.11*** | -26.78*** |  | -38.08*** | -34.60*** |  | -24.80*** | -24.64*** |
|  | (43.41) | (44.62) |  | (6.91) | (6.84) |  | (11.35) | (10.74) |  | (5.71) | (6.54) |
| Squared Farm Size (ha) | 11.90 | 5.56 |  | 0.70*** | 0.60*** |  | 1.73*** | 1.59*** |  | 0.64*** | 0.53*** |
|  | (8.44) | (8.74) |  | (0.22) | (0.21) |  | (0.58) | (0.54) |  | (0.19) | (0.20) |
| Age HH head (years) | 0.84 | 1.02 |  | 1.23 | 1.31 |  | 0.99 | 1.19 |  | 1.20 | 1.34 |
|  | (1.00) | (1.04) |  | (0.92) | (0.94) |  | (0.92) | (0.94) |  | (0.86) | (0.87) |
| Household size | 7.66 | 3.90 |  | 5.03 | 1.93 |  | 6.12 | 2.44 |  | 4.56 | 1.76 |
|  | (5.59) | (5.77) |  | (5.05) | (5.15) |  | (5.00) | (5.13) |  | (4.63) | (4.71) |
| =1 if female headed HH | -46.85 | -69.35 |  | -53.35 | -70.40* |  | -41.74 | -60.89 |  | -47.48 | -62.51* |
|  | (39.49) | (42.19) |  | (36.70) | (39.22) |  | (36.34) | (38.43) |  | (34.47) | (36.67) |
| Years of education of HH head | -3.07 | -4.33* |  | -2.25 | -3.42 |  | -2.72 | -3.68* |  | -2.28 | -3.09 |
|  | (2.45) | (2.38) |  | (2.31) | (2.21) |  | (2.31) | (2.23) |  | (2.17) | (2.07) |
| =1 if Ogun state | 85.12** | -106.50 |  | 80.78** | -107.02* |  | 86.09** | -94.43 |  | 83.34*** | -97.90 |
|  | (37.16) | (71.39) |  | (33.24) | (64.53) |  | (34.04) | (67.48) |  | (31.08) | (61.64) |
| Family labor days/ha |  | 0.85* |  |  | 0.91*** |  |  | 0.86** |  |  | 0.84** |
|  |  | (0.45) |  |  | (0.35) |  |  | (0.40) |  |  | (0.34) |
| Hired labor days/ha |  | 0.06 |  |  | -0.11 |  |  | -0.75 |  |  | -0.65 |
|  |  | (1.33) |  |  | (0.70) |  |  | (0.58) |  |  | (0.45) |
| Fertilizer (kg/ha) |  | -0.14 |  |  | -0.12 |  |  | -0.13 |  |  | -0.11 |
|  |  | (0.19) |  |  | (0.17) |  |  | (0.18) |  |  | (0.17) |
| Dummy variables (access to market, extensions, agro-dealer) | Yes | Yes |  | Yes | Yes |  | Yes | Yes |  | Yes | Yes |
| Dummy variables for crop categories (grains, legumes, roots & tubers, fruits & vegetables, cash crops) |  | Yes |  |  | Yes |  |  | Yes |  |  | Yes |
| HH assets (radio, TV, mobile phone, car, motorcycle) | Yes | Yes |  | Yes | Yes |  | Yes | Yes |  | Yes | Yes |
| Farm equipment (water pump, tractor, sprayer) | Yes | Yes |  | Yes | Yes |  | Yes | Yes |  | Yes | Yes |
| Soil quality & types |  | Yes |  |  | Yes |  |  | Yes |  |  | Yes |
| Dummy variables for Local Government Areas (LGAs) | Yes | Yes |  | Yes | Yes |  | Yes | Yes |  | Yes | Yes |
| =1 if HH ‘stepped up’ from small-scale |  |  |  |  |  |  |  |  |  |  | -145.56*** |
|  |  |  |  |  |  |  |  |  |  |  | (39.10) |
| ‘Stepped up’*farm size interaction term |  |  |  |  |  |  |  |  |  |  | 19.75*** |
|  |  |  |  |  |  |  |  |  |  |  | (4.49) |
| Constant | 205.71*** | 292.82** |  | 133.73** | 299.26*** |  | 152.61*** | 236.35** |  | 133.74** | 274.52*** |
|  | (79.44) | (128.57) |  | (57.16) | (104.31) |  | (56.95) | (103.12) |  | (54.15) | (96.38) |
| Observations | 1,103 | 1,103 |  | 1,637 | 1,637 |  | 1,565 | 1,565 |  | 2,079 | 2,079 |
| R-squared | 0.11 | 0.16 |  | 0.10 | 0.15 |  | 0.09 | 0.14 |  | 0.09 | 0.14 |
| Turning point for cultivated farm size (ha) |  |  |  | 20.8 | 22.1 |  | 11.0 | 10.9 |  | 19.50 | 23.3 |
| Turning point for cultivated farm size based on stepping up (ha) |  |  |  |  |  |  |  |  |  |  | 7.4 |

Robust standard errors in parentheses. *** p<0.01, ** p<0.05, * p<0.1

Notes: ^*^Model II comprises small and medium-scale farmers that stepped into medium-scale farming with no prior experience as small-scale farmers (stepped-in)

^+^Model III comprises small-scale and medium-scale farmers that stepped into medium-scale farming with prior experience as small-scale farmers (stepped-up)

^◊^Model IV is the full sample including small and medium-scale farmers with stepped-in and stepped-up farmers inclusive.
